# Next‐Generation CAR‐T Cell Therapy: From Modular Design to Clinical Expansion

**DOI:** 10.1002/exp2.70230

**Published:** 2026-10-21

**Authors:** Yuesong Bai, Jintao Qin, Zongjun Dai, Ran Yan, Yong Yang, Wenguang Wang

**Affiliations:** ^1^ Institute of Translational Medicine China Pharmaceutical University Nanjing China; ^2^ Center for New Drug Safety Evaluation and Research China Pharmaceutical University Nanjing China; ^3^ School of Biopharmacy China Pharmaceutical University Nanjing China; ^4^ Key Laboratory of Biomedical Functional Materials School of Science China Pharmaceutical University Nanjing China

**Keywords:** autoimmune disorders, cancer, CAR‐T cell therapy, dynamic immune engineering

## Abstract

Chimeric antigen receptor (CAR)‐T cell therapy has emerged as a groundbreaking treatment for cancer. After decades of research, it is now being actively explored for a broader range of immune‐mediated diseases, particularly cancer and autoimmune disorders. Significant progress has been made in both understanding technological advancements and addressing the intrinsic limitations of this engineered cellular therapy. However, the clinical efficacy and safety of CAR‐T cells remain influenced by several factors, including the immunosuppressive tumour microenvironment (TME), the continuous mutational evolution of tumour cells, the prohibitive cost of production limiting accessibility, and the immune‐related toxicities associated with biologic treatments. Given these substantial challenges, a paradigm shift is urgently needed – from conceptualizing CAR‐T cells solely as “engineered immune cells” to recognizing them as “programmable intelligent systems.” This review proposes a dynamic immune engineering framework, advocating that the next generation of CAR‐T cells be conceived as programmable immune systems capable of sensing, responding to, and self‐regulating immune responses. This evolving approach positions dynamic immune regulation as the core principle underlying immune system function. Ultimately, this review aims to provide a conceptual and theoretical foundation for the development of next‐generation CAR‐T therapies that are not only highly effective but also cost‐efficient and globally accessible.

## Graphical abstract

Over the past decades, chimeric antigen receptor (CAR)‐T cell therapy has evolved from a linear engineering concept into a powerful yet constrained immunotherapy, limited by tumour microenvironment barriers, immune evasion, and manufacturing complexity. This review proposes a dynamic immune engineering framework that redefines CAR‐T cells as adaptive, programmable systems, enabling broader, safer, and more accessible applications across cancer and autoimmune diseases.

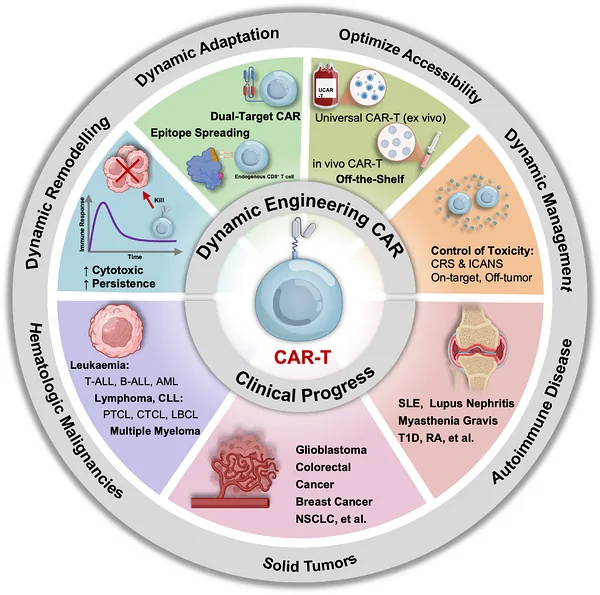

## 1 Introduction

Chimeric antigen receptor (CAR)‐T cell therapy has emerged as one of the most transformative advances in cancer immunotherapy, marking a paradigm shift in the treatment of haematologic malignancies. By engineering autologous T cells to express synthetic receptors that recognize tumour‐associated antigens (TAAs) independent of major histocompatibility complex (MHC) presentation [1], CAR‐T therapy circumvents key limitations of conventional immunotherapies and has demonstrated remarkable clinical efficacy, particularly in haematological malignancies such as relapsed/refractory B cell acute lymphoblastic leukaemia (R/R B‐ALL), large B cell lymphoma (LBCL), and multiple myeloma (MM) [2, 3, 4]. The successive regulatory approvals of CD19‐ and B‐cell Maturation Antigen (BCMA)‐targeting CAR‐T products by agencies including the US FDA, EMA, and China's NMPA have catalysed a new era of precision cellular immunotherapy.

Over the past four decades, CAR‐T cell therapy has evolved from a conceptual framework into a robust therapeutic platform. From first‐generation CARs with limited in vivo activity to second‐ and third‐generation CARs that achieve greater clinical efficacy through incorporation of costimulatory domains, and further to fourth‐generation designs featuring safety switches, cytokine armouring, or logic‐gated circuits, the field has undergone substantial innovation. This technological progress has not only improved T cell persistence and functionality but also broadened the scope of treatable malignancies [5, 6]. Recently, CAR‐T cell therapy has achieved remarkable progress. However, its application in oncology has also revealed substantial and concerning limitations, such as tumour antigen escape, T cell exhaustion, and restricted expansion in immunosuppressive environments [7, 8, 9, 10]. Numerous high‐quality reviews have comprehensively addressed these issues in structural optimization, target selection, and clinical management [11, 12]. However, the aspirational goal of achieving “one infusion, lifelong benefit” a critical reality is often overlooked: once administered, CAR‐T cells function as living, evolving therapeutic entities whose performance is continuously reshaped by the host immune ecosystem. Factors such as tumour microenvironment (TME) pressure, intercellular interactions, and immunosuppressive signalling dynamically modulate their therapeutic activity [13, 14, 15]. Moreover, considerations of manufacturing costs and therapeutic accessibility should be integrated into the framework of future innovation [9]. Under these circumstances, the inherent complexity of the TME and immune ecology dictates that future CAR‐T designs must transcend the traditional “construct–infuse–observe” paradigm. Instead, they should adopt an integrated approach encompassing design, administration, and post‐treatment adaptation, enabling a “sense–respond–self‐regulate” closed‐loop model – an intelligent immunological automaton capable of real‐time adaptation within the patient.

In response to these challenges, a new generation of design strategies has emerged to redefine the functional and structural paradigm of CAR‐T therapy. These strategies include modulating CAR signalling pathways through genetic engineering [16, 17], as well as selecting T cell subsets with greater proliferative potential and sustained activity – such as stem cell–like memory T cells (T_SCM_) – to remodel the TME and prolong therapeutic efficacy [18]. In addition, dual‐ or multi‐target CAR constructs have been designed [19, 20], together with interactions with antigen‐presenting cells to induce epitope spreading (ES) [21], thereby enabling dynamic antigen adaptation, mitigating immune escape, and broadening tumour coverage. Meanwhile, non‐viral transduction strategies and universal allogeneic CAR‐T products have become research hotspots, aiming to reduce costs, simplify manufacturing, improve biosafety and accessibility, and ultimately establish an “off‐the‐shelf” therapeutic model. Furthermore, real‐time monitoring during treatment and follow‐up, combined with the use of monoclonal antibodies and cytokines, is being explored to achieve dynamic toxicity control and mitigate the unique immune‐related adverse events associated with CAR‐T as a biological product [10, 22, 23].

In parallel with these design innovations, the clinical landscape of CAR‐T therapy has undergone rapid expansion. While initial approvals were confined to haematologic malignancies, clinical trials are now actively exploring CAR‐T applications in a wide array of solid tumours, including glioblastoma (GBM), colorectal cancer (CRC), and hepatocellular carcinoma (HCC) [24, 25, 26]. Moreover, recent studies have begun to explore CAR‐T therapies beyond oncology in autoimmune diseases (AIDs) such as systemic lupus erythematosus (SLE) and dermatomyositis, demonstrating the ability to eliminate pathogenic autoreactive clones and to re‐establish immune tolerance, thereby mitigating disease progression (PD) [27]. These emerging indications further underscore the versatility and adaptability of CAR‐based cellular therapies.

This review provides a comprehensive overview of the developmental trajectory and cutting‐edge technological advances of CAR‐T cell therapy, with a particular emphasis on recent breakthroughs in structural design that address four critical challenges: tumour microenvironmental suppression, immune evasion, manufacturing bottlenecks, and treatment‐related toxicities. We highlight how these innovations collectively position the next generation of CAR‐T products as “intelligent immune automaton” capable of real‐time adaptation within dynamic immune ecosystems. In addition, we summarize the latest clinical progress of CAR‐T therapies in haematologic malignancies, solid tumours, and AIDs, underscoring their therapeutic potential as the field expands from oncology into broader immune disorders. Finally, we discuss the major barriers that hinder wider clinical translation and dissemination and provide a forward‐looking perspective on future directions, aiming to offer a comprehensive reference and strategic insight for the continued innovation and clinical advancement of CAR‐T therapies.

## 2 40 Years of CAR‐T: A New Paradigm Beyond Conventional Linear Models

The origin of CAR‐T cell therapy dates back to the late 1980s, when researchers began exploring synthetic receptors to endow T cells with tumour specificity. In 1987, Kurosawa's team first fused the variable region of an immunoglobulin with the constant region of the T cell receptor (TCR), generating a chimeric TCR that relied on MHC‐independent recognition and demonstrating the activation potential of engineered T cells [28]. Two years later, Eshhar and colleagues at the Weizmann Institute recombined antibody variable domains (VH/VL) with the TCR constant region, which enabled T cells to recognize tumour antigens independently of MHC, laying the theoretical foundation for modern CAR‐T development (Figure 1) [29].

**FIGURE 1:**
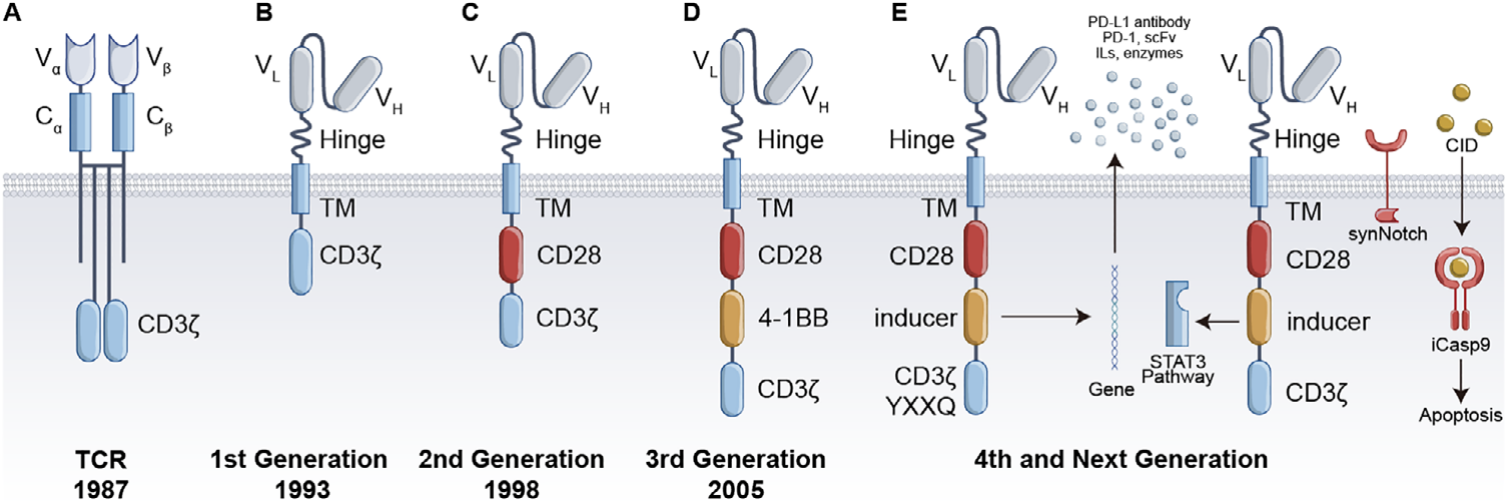
The structural evolution of CAR‐T cells from TCR to next‐generation CARs. (A) Schematic of the native TCR complex (1987). Its full activation is mediated by the synergistic integration of antigen presentation in an MHC‐dependent manner and costimulatory signalling. (B) First‐generation CAR‐T cells (1993) contain an scFv for antigen recognition, a hinge/transmembrane domain, and a CD3ζ signalling domain, allowing MHC‐independent activation but exhibiting limited persistence. (C) Second‐generation CAR‐T cells (1998) incorporate one costimulatory domain (e.g., CD28 or 4‐1BB), enhancing proliferation, survival, and cytotoxicity. (D) Third‐generation CARs (2005) integrate dual costimulatory domains (e.g., CD28 and 4‐1BB), further augmenting metabolic fitness and resistance to exhaustion. (E) Fourth‐generation and next‐generation CARs (e.g., TRUCKs, logic‐gated CARs, and armoured CARs) are equipped with inducible cytokine expression, TME‐modulating functions, and built‐in safety switches, representing a new level of therapeutic sophistication.

In the early 1990s, single‐chain variable fragments (scFv) gradually replaced traditional antibody formats, and their fusion with the CD3ζ chain of the TCR gave rise to the first‐generation CARs. These constructs enabled stable transduction into primary T cells using a single vector system [30]. However, these first‐generation CARs, relying solely on CD3ζ signalling, exhibited limited activation efficiency and poor in vivo persistence [31]. The emergence of the “two‐signal theory” for T cell activation subsequently paved the way for second‐generation CARs [32, 33, 34]. In 1997, Cell Genesys Inc. filed patents related to the incorporation of costimulatory domains [35]. In 1998, Sadelain's team developed a CAR structure consisting of scFv‐CD28‐CD3ζ, marking the maturation of second‐generation CARs [36]. CD28 signalling activates the PI3K/AKT pathway, significantly enhancing T cell proliferation and survival, resulting in superior antigen‐dependent expansion, IL‐2 production, and longer in vitro persistence – features absent in first‐generation CARs, which lack costimulatory signals [36, 37, 38]. In 2011, Carl June's team achieved durable remission in patients with R/R CLL, leading to the US FDA approval of tisagenlecleucel (Kymriah) in 2017, the first CAR‐T product on the market [39].

However, dose‐dependent toxicities such as cytokine release syndrome (CRS) and antigen escape highlighted the limitations of relying on a single costimulatory signal, which led to the development of third‐generation CARs. By integrating multiple costimulatory domains (e.g., CD28 and 4‐1BB), third‐generation CARs exhibit enhanced metabolic adaptability and anti‐apoptotic capacity [40, 41]. However, immunosuppressive factors within the TME and signalling redundancy continued to limit efficacy, particularly in solid tumours [42]. To address these issues, fourth‐generation CARs introduced “smart” designs, such as the Synthetic Notch (SynNotch) system enabling dual‐antigen logic‐gated recognition, the T cells redirected for antigen‐unrestricted cytokine‐initiated killing (TRUCKs) platform promoting local cytokine (e.g., IL‐12) release to remodel the TME, and the iCasp9 suicide switch for improved safety control [43, 44, 45, 46, 47]. Notably, armoured CARs targeting IL‐13Rα2 showed promising results in GBM models, demonstrating potential in treating solid tumours [48]. Although CAR‐T therapy has shown limited efficacy in certain solid tumours, its clinical performance remains significantly constrained by T cell exhaustion, heterogeneity, and antigen escape, as well as physical barriers within the TME [10, 49]. These challenges have driven the advancement toward next‐generation CARs, aiming to develop systems with dynamically programmable functions for precise regulation and sustained responses within complex tumour settings.

## 3 From Static Design to Dynamic Regulation: A New Framework for CAR‐T

Despite the remarkable clinical success and widespread recognition of approved CAR‐T therapies in haematologic malignancies, their efficacy is hindered by a range of dynamic challenges [50, 51, 52, 53]. These include insufficient cytotoxicity, PD induced by antigen escape, and immunosuppression mediated by the TME. Mechanistically, the functional attenuation of CAR‐T cells stems from both intrinsic and extrinsic pressures faced within a dynamic tumour environment [13, 42]. Intrinsically, prolonged antigen exposure induces T cell exhaustion, a state of dynamic cellular dysfunction characterized by diminished cytokine secretion and the upregulation of inhibitory receptors like PD‐1 and apoptosis‐related markers like Fas [54]. Extrinsically, suppressive cues within the TME – including regulatory T cells (Tregs), myeloid‐derived suppressor cells (MDSCs), inhibitory cytokines (e.g., TGF‐β), metabolic deprivation, and checkpoint ligands such as PD‐L1 – further dynamically compromise CAR‐T cell infiltration, persistence, and cytotoxicity [55, 56, 57, 58, 59]. To overcome these multifactorial and dynamically changing barriers, current research is focused on driving technological innovations to restore and sustain CAR‐T cell efficacy through integrated strategies such as cellular engineering, immune modulation, and combination therapies. This paradigm shift, from “static design” to “dynamic regulation”, is the core direction for the future of CAR‐T design and development.

### 3.1 Dynamic Remodelling of the TME: Unleashing Function From Gene to Cell

#### 3.1.1 Strategies to Overcome CAR‐T Functional Attenuation and Identify Intervention Pathways

The therapeutic efficacy of CAR‐T cells in vivo is frequently compromised by limited cytotoxic activity, functional exhaustion, and premature apoptosis, particularly within a hostile TME [60]. To address these challenges, contemporary strategies seek to augment CAR‐T cell cytotoxicity and survival via genetic engineering of effector molecules, modulation of apoptotic and checkpoint pathways, and transcriptional reprogramming [17, 61, 62]. Simultaneously, reshaping the immunosuppressive tumour milieu – such as targeting tumour‐associated macrophages (TAMs) – further boosts CAR‐T performance [63]. Collectively, these approaches aim to maximize CAR‐T potency and persistence in vivo, thereby ensuring durable and effective antitumour responses.

##### 3.1.1.1 Enhancing the Cytotoxic Activity of CAR‐T Cells

To augment the cytotoxic activity of CAR‐T cells, genetic engineering approaches have been widely utilized to precisely regulate cytolytic effector functions. CAR‐T cells induced apoptosis in target cells through programmed cell death pathways, with the Fas/FasL axis playing a pivotal role [54]. Through this pathway, CAR‐T cells triggered apoptosis in tumour cells, thereby enhancing cytotoxic efficacy [54]. Notably, the Fas/FasL axis also mediates bystander killing of antigen‐negative tumour cells, thereby broadening the cytotoxic spectrum of CAR‐T therapy [61].

However, recent studies have uncovered key limitations of Fas/FasL‐mediated bystander killing within the solid tumour TME. Specifically, a P153 (Proline → Serine) point mutation in human FasL markedly increases its susceptibility to plasmin‐mediated cleavage, resulting in rapid inactivation of membrane‐bound FasL and diminished apoptotic activity [64]. Given that proteases such as plasmin are often upregulated in solid tumours, this cleavage mechanism can substantially impair FasL‐mediated cytotoxicity. To overcome this obstacle, investigators have engineered scFv antibodies (e.g., Nok2‐FasL) that selectively mask the cleavage site without interfering with Fas binding, thereby preserving the cytotoxic function of FasL. Further investigations have confirmed that membrane‐bound FasL promotes greater Fas receptor clustering and killing capacity relative to its soluble form, underscoring the importance of maintaining membrane anchorage for optimal FasL functionality [64]. These findings identify a bottleneck in FasL‐mediated cytotoxicity in solid tumours and suggest potential engineering strategies – such as enhancing FasL membrane anchorage and protease resistance – to restore efficacy [65]. Moreover, a novel NeoCAR‐T targeting KRAS G12V mutations – integrating IL‐12 secretion and TCR knockout – has exhibited enhanced efficacy and safety across multiple solid‐tumour models. Taken together, these data highlight the therapeutic promise of multi‐layered genetic engineering strategies to synergistically optimize CAR‐T cell performance [16].

Beyond genetic engineering of CAR‐T cells themselves, modulating the TME to alleviate immunosuppression has emerged as a complementary strategy to enhance CAR‐T cytotoxicity. Studies have shown that the selective nuclear export inhibitor Selinexor (SEL) can suppress the polarization of TAMs toward the immunosuppressive M2 phenotype, thereby significantly alleviating the suppressive nature of the TME and enhancing the anti‐lymphoma efficacy of CAR‐T cells against B cell malignancies [63]. In multiple ex vivo and in vivo models, SEL not only decreases the proportion of M2‐type macrophages but also upregulates the expression of NGFR in CAR‐T cells, promoting their expansion and functional enhancement. Although high‐dose SEL in combination therapy may induce adverse effects (AEs) such as elevated TNF‐α levels and anaemia, these toxicities are mitigated by adjusting dose and schedule, such as administering low‐dose SEL after platelet recovery – thus achieving a balance between efficacy and safety [63]. Therefore, these findings suggest that targeting immunoregulatory cells within the TME is a promising adjunct to CAR‐T therapy, particularly in tumours characterized by highly immunosuppressive milieus.

##### 3.1.1.2 Enhancing CAR‐T Cell Persistence

Sustaining CAR‐T cell persistence is a major research focus. Researchers have employed CRISPR/Cas9 to knock out the PD‐1 gene, resulting in significantly enhanced cytokine secretion, multifunctionality, in vivo antitumour activity of low‐affinity CAR‐T cells, and reduced expression of exhaustion‐related genes. These findings suggest that PD‐1 disruption reverses the dysfunctional state of low‐affinity CAR‐T cells, thereby improving their therapeutic efficacy [17]. To achieve more precise control, researchers have developed innovative ‘switch receptor’ designs. For instance, by fusing the extracellular domain of PD‐1 to the intracellular signalling domain of CD28, they constructed a PD‐1:CD28 chimeric receptor. This design converts the PD‐L1/PD‐1 inhibitory signal into a costimulatory signal, enabling CAR‐T cells to become activated rather than suppressed upon encountering PD‐L1‐expressing tumour cells [66]. This strategy has been validated in solid tumour models, showing enhanced T cell infiltration, prolonged functional persistence, and improved antitumour activity, culminating in more effective tumour clearance. Furthermore, systematic screening using various CRISPR‐based platforms has led to the identification of novel regulators of CAR‐T cell persistence, including RHOG, RASA2, and SOCS1, whose roles have been further validated in in vivo models [67, 68].

In addition, modulating transcription factors that influence T cell fate decisions has emerged as a key approach to sustaining CAR‐T functionality. In 2019, Lynn et al. demonstrated that overexpression of c‐Jun suppressed the exhaustion program, enabling CAR‐T cells to maintain high IFN‐γ levels during long‐term proliferation [69]. Meanwhile, in animal models, simultaneous knockout of PRDM1 (BLIMP1) and NR4A3 enriches T_SCM_ populations, significantly improving the durability of CAR‐T responses [70]. These transcription factor–based strategies enhance CAR‐T persistence and offer a mechanistic framework for sustaining antitumour efficacy under chronic antigen stimulation. Moreover, researchers have utilized viral immune evasion tactics to boost the in vivo persistence of CAR‐T cells. By utilizing the HIV‐1 Nef protein, allogeneic CAR‐T cells were engineered to downregulate HLA‐I expression, evading CD8^+^ T cell recognition while preventing nature killer (NK) cell activation. Importantly, the degree of HLA‐I downregulation was finely tuned to avoid NK cell‐mediated lysis, balancing immune evasion with safety. Nef also activates the Pak2 signalling pathway, inducing the expression of the anti‐apoptotic protein B‐cell lymphoma‐2 (BCL‐2), which suppresses activation‐induced cell death (AICD) in CAR‐T cells. This prolongs CAR‐T cell survival and boosts antitumour efficacy. This strategy offers a novel molecular target and approach to improve the durability and functional stability of CAR‐T cell therapies [71]. In the most recent study, CAR constructs incorporating a ZAP327 signalling domain have shown stronger in vivo expression at both day 3 and day 7 compared with conventional CAR‐T cells [72]. This strategy has been associated with enhanced enrichment of T_SCM_, reduced exhaustion and cytokine production, resulting in prolonged CAR‐T persistence and improved antitumour responses and safety during treatment.

In addition to targeting exhaustion signalling, regulating apoptotic pathways is another critical strategy to enhance CAR‐T cell persistence. Although the Fas/FasL pathway can mediate the programmed death of target cells, Fas is also expressed on the surface of CAR‐T cells, making them susceptible to “suicide” induced by FasL derived from tumour cells or neighbouring T cells [65, 73]. Previous studies have engineered Fas‐TNFR chimeric receptors (e.g., Fas‐CD40) to block Fas‐mediated apoptotic signalling, which significantly enhances the proliferative capacity and antitumour efficacy of CAR‐T cells [74]. Furthermore, by constructing Fas dominant‐negative receptor (Fas DNR) variants to block Fas signalling, FasL‐induced AICD can be effectively suppressed, thereby enhancing CAR‐T cell survival and functional persistence within the TME [75]. Altogether, enhancing CAR‐T persistence requires inhibiting exhaustion and apoptotic pathways, along with reprogramming T cell fate at the molecular level. From PD‐1 disruption to synthetic switch receptor rewiring inhibitory signals and incorporating viral immune evasion strategies to promote survival, these approaches reflect a paradigm shift beyond conventional therapies. Moving forward, the key challenge will lie in balancing enhanced resilience and sustained functionality with precise control over activation thresholds and safety – paving the way for CAR‐T products that are both powerful and precisely regulated.

### 3.2 Immune Cell Suppression in the TME

The TME remains a significant barrier to effective CAR‐T cell infiltration and cytotoxicity in solid tumours due to its complex network of cellular interactions that not only support tumour survival and growth but also promote immune evasion, allowing tumours to escape immune surveillance and resist therapeutic interventions (Figure 2) [76, 77]. Within this immunosuppressive environment, dynamic interactions among MDSCs, Tregs, and tumour cells critically contribute to tumour progression and CAR‐T cell dysfunction.

**FIGURE 2:**
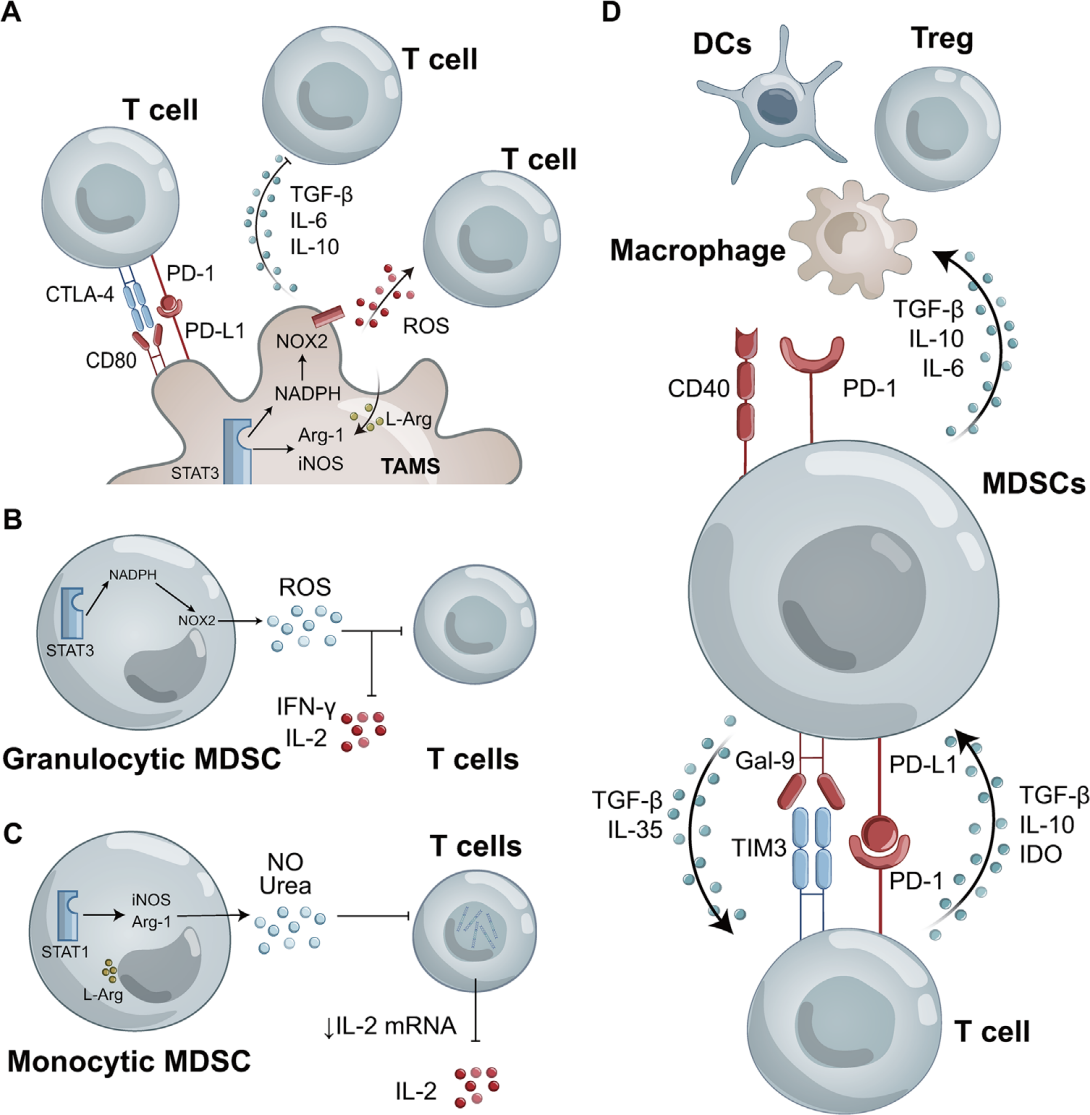
Mechanisms by which immunosuppressive components in the TME contribute to tumour progression. Immunosuppressive cell populations in the TME, including TAMs, MDSCs, and Tregs, cooperate to suppress effector T cell function through multiple mechanisms, thereby promoting tumour growth and immune evasion. (A) Three immunosuppressive mechanisms of TAMs on CTLs. Chemotactic recruitment: TAMs suppress CTL function by expressing PD‐L1 and CD80, which bind to PD‐1 and CTLA‐4 on CTLs, and by secreting immunosuppressive cytokines such as IL‐10, TGF‐β, and IL‐6. Induction of immunosuppressive mediators: TAMs secrete ROS, IL‐10, and TGF‐β, enhancing their M2 polarization and promoting the production of arginine‐metabolizing enzymes such as Arginase 1 (Arg‐1) and iNOS, thereby further suppressing T cell activity. Cell‐contact‐dependent synergy: CTLs directly reinforce the suppressive phenotype of TAMs via CTLA‐4‐mediated contact‐dependent signalling, maintaining the immunosuppressive tumour microenvironment. (B) Suppression of T cell function by granulocytic G‐MDSCs. G‐MDSCs activate NADPH oxidase (NOX2) through the STAT3 signalling pathway, generating reactive oxygen species (ROS) that inhibit the production of key activation factors by T cells, such as interferon‐γ (IFN‐γ) and interleukin‐2 (IL‐2), reducing their antitumour activity. (C) Metabolic suppression mechanisms of monocytic MDSCs (M‐MDSCs). M‐MDSCs induce expression of iNOS and Arg‐1 via the STAT1 pathway, which metabolize L‐arginine into nitric oxide (NO) and urea; NO inhibits IL‐2 mRNA expression in T cells, thus blocking their proliferation and functional activation. (D) Promotion of immunosuppression and inhibition of T cell–mediated tumour killing by MDSCs and Tregs. MDSCs suppress cytotoxic T lymphocytes (CTLs) and promote the conversion of conventional T cells into Tregs by secreting immunosuppressive cytokines such as TGF‐β, IL‐6, and IL‐10. Conversely, Tregs can also enhance the immunosuppressive phenotype of MDSCs. These two cell types interact through co‐inhibitory molecules such as PD‐1 and TIM‐3, establishing a stable immunosuppressive network. MDSCs also inhibit the maturation of dendritic cells and macrophages by releasing immunomodulatory molecules, including TGF‐β, IL‐10, and indoleamine 2,3‐dioxygenase (IDO), and reduce T cell activity by expressing co‐inhibitory ligands such as PD‐L1, CD80, and CD40. In addition, immunosuppressive effects are further amplified through pathways involving IL‐35 and TGF‐β, enabling MDSCs to cooperate with Tregs in maintaining immune tolerance within the tumour microenvironment.

#### 3.2.1 MDSC and Treg

MDSCs are key components of the TME, playing critical roles in regulating the progression and metastasis of solid tumours. MDSCs exert immunosuppressive effects by secreting pleiotropic cytokines (e.g., TGF‐β1, IL‐10, IL‐6) and depleting amino acids, such as arginine and tryptophan, which are critical for T cell activation and survival. These actions are crucial in suppressing T cell proliferation, differentiation, and effector functions while promoting the generation of Tregs [78, 79, 80, 81, 82]. The TME also includes immune checkpoints such as PD‐1/PD‐L1, LAG‐3, CTLA‐4, and TIM‐3, along with infiltrating immunosuppressive myeloid cells and Tregs, collectively suppressing endogenous antitumour immune responses and impairing the therapeutic efficacy of CAR‐T cells [83]. Previous studies have shown that deletion of Triggering Receptor Expressed on Myeloid Cells 2 (TREM2) on myeloid cells enhances the efficacy of anti–PD‐1 immunotherapy [84]. Building on this, Jian Chen and colleagues developed a CRC therapy in which CAR‐T cells were genetically engineered to secrete a bispecific PD‐1/TREM2 scFv. In a subcutaneous CRC mouse model, these CAR‐T cells demonstrated superior tumour elimination compared to CAR‐T cells secreting PD‐1 scFv alone. Moreover, the PD‐1‐TREM2 scFv secreted by the CAR‐T cells was retained within the tumour and exhibited an extended half‐life [85].

Tregs play a pivotal role in mediating immune suppression and facilitating tumour immune evasion [86]. Within the TME, excessive accumulation of Tregs can negatively impact the antitumour activity of CAR‐T cells [87]. This is particularly evident in solid tumours, where Treg infiltration may mediate immunosuppression through the CTLA‐4 receptor, reducing the cytotoxic efficacy of CAR‐T cells [87, 88]. On one hand, Tregs play a crucial role in suppressing excessive immune responses, such as those seen in AIDs [89]; on the other hand, their immunosuppressive function within the TME may hinder the effective response of CTLs against cancer cells [90].

#### 3.2.2 Selecting T Cell Subsets to Overcome the TME Barrier

Different T cell subsets possess distinct characteristics, and by leveraging the unique properties of each subset, researchers can significantly enhance the cytotoxicity and persistence of CAR‐T cells. This intercellular cooperation – mediated by cytokine‐based communication – contributes to more effective antitumour responses within the TME [14, 91]. Both CAR‐engineered CD8^+^ and CD4^+^ T cells have demonstrated potent tumour‐lytic activity in various animal models [92, 93]. Among them, CAR CD8^+^ T cells primarily mediate contact‐dependent cytotoxicity [14], whereas CAR CD4^+^ T cells support tumour elimination by assisting endogenous effector cells and releasing cytokines such as IFN‐γ and TNF‐α [94, 95]. Moreover, studies have shown that CD8^+^ and CD4^+^ T cells exert synergistic antitumour effects in the TME. Through IFN‐γ and IL‐12 signalling, CAR‐T cells interact with the TME to modulate cytotoxic functions [14]. In animal models, a 1:1 ratio of CAR CD8^+^ and CAR CD4^+^ T cells achieved superior therapeutic responses compared to unsorted CAR‐T cells or single‐subtype CAR CD8^+^/CAR CD4^+^ therapies [92]. Clinical data also confirm this cooperative effect using a 1:1 CD4^+^/CD8^+^ CAR‐T cell ratio; the complete remission (CR) rate reached 96%, with 86% achieving minimal residual disease (MRD) negativity in a low‐dose treatment cohort of adult B‐ALL patients [96]. This finding underscores the clinical relevance of maintaining an optimal CD4^+^/CD8^+^ ratio.

The differentiation status of T cells also plays a critical role in CAR‐T therapy. Compared to effector memory T cells (T_EM_) and effector T cells (T_EFF_), less differentiated subsets – such as naïve T cells (T_N_), T_SCM_, and central memory T cells (T_CM_) – often exhibit superior engraftment potential, persistence, and antitumour immunity [91, 97]. In animal models, studies have shown that CAR‐T cells generated from purified T_N_ and T_SCM_ populations demonstrate more durable and potent antitumour activity [98]. Clinical evidence further supports this, indicating that therapeutic outcomes correlate with the relative abundance of specific T cell subsets following infusion. Patients who achieved CR exhibited higher proportions of T_SCM_ and T_CM_ compared to those who experienced relapse [99].

In recent years, double‐negative T cells (DNTs; CD3^+^CD4^−^CD8^−^) have emerged as a novel subset with promising antitumour properties. Unlike conventional CD4^+^ and CD8^+^ T cells, DNTs do not rely on TCR‐mediated antigen recognition and possess intrinsic antitumour activity along with low alloreactivity. When engineered with second‐generation CARs, DNT‐based CAR‐T cells did not induce graft‐versus‐host disease (GvHD) in allogeneic transplantation models and demonstrated tumour control comparable to that of traditional CAR‐T cells, highlighting their potential as an “off‐the‐shelf” cell therapy platform [100]. These findings underscore the pivotal impact of T cell subset selection and differentiation status on the efficacy of CAR‐T therapy. They suggest that a one‐size‐fits‐all approach may not be adequate for clinical needs. Instead, tailoring T cell composition according to tumour type and its immunosuppressive microenvironment may be key to unlocking the full therapeutic potential of CAR‐T cells.

### 3.3 Dynamic CAR‐T Adaptation: Multi‐Antigen Synergy to Counter Immune Evasion

Antigen heterogeneity in tumour cells results in antigen‐negative subpopulations in vivo, enabling these cells to evade CAR‐T cell recognition (Figure 3) [101, 102]. A critical challenge for CAR‐T therapy lies in the selection of an ideal target antigen, which is typically classified as either a TAA or a tumour‐specific antigen (TSA) [42]. TAAs are proteins that are overexpressed on tumour cells but are also present at low levels on healthy cells. Because they are abundant in many types of cancer, they are frequently chosen as CAR‐T targets, but their presence on healthy tissues carries the risk of “on‐target, off‐tumour (OTOT)” toxicity, where CAR‐T cells mistakenly attack healthy organs [40]. In contrast, TSAs are ideal targets, as they are uniquely expressed on cancer cells and absent from healthy tissues [103]. These antigens are typically derived from neo‐epitopes created by tumour‐specific mutations or from viral antigens in virally induced cancers [104]. The unique nature of TSAs offers high specificity and safety, as attacking them will not harm healthy cells. However, a major hurdle in CAR‐T development, especially for solid tumours, is the scarcity of identified TSAs that are both stable and universally present across a patient's tumour [42]. Consequently, most current CAR‐T therapies are forced to target TAAs.

**FIGURE 3:**
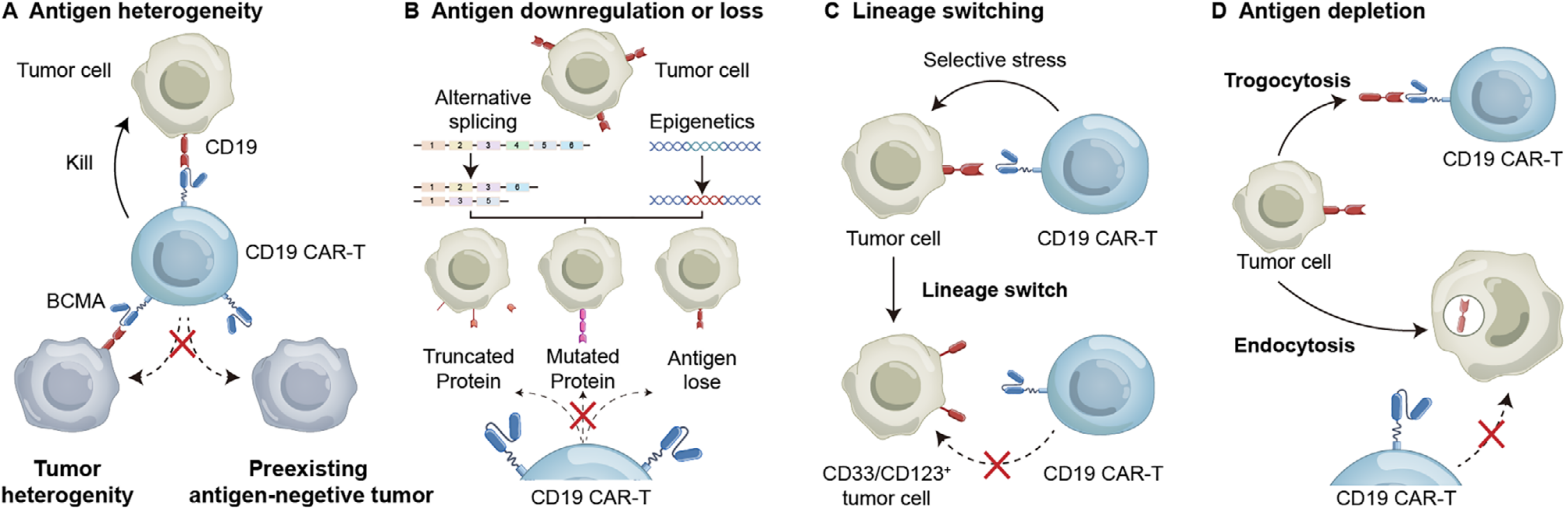
Tumour cells can evade CAR‐T therapy through multiple mechanisms of antigen escape. (A) Antigen heterogeneity: Certain tumour cells inherently lack expression of CAR‐T target antigens (e.g., CD19) or express non‐specific antigens, thereby evading CAR‐T‐mediated immune clearance. (B) Antigen downregulation or loss: Under therapeutic pressure, tumour cells may downregulate or lose target antigen expression through genetic mutations, alternative splicing, or epigenetic modifications, resulting in the failure of CAR‐T cell recognition. (C) Lineage switching: Some malignancies, including B cell leukaemia, may undergo conversion to myeloid phenotypes (e.g., CD33^+^/CD123^+^) during treatment, thus evading CD19‐directed CAR‐T recognition. (D) Antigen depletion: Surface antigens can be lost through CAR‐T‐induced trogocytosis or internalization by tumour cells, reducing recognition and cytotoxic activity.

This dynamic landscape makes a single‐target CAR‐T, designed to attack a specific TAA or TSA, vulnerable to antigen escape. This can occur when a tumour cell dynamically loses, downregulates, or alters its antigen expression through genetic mechanisms like mutation and selective regulation [105, 106, 107, 108]. Additionally, cellular processes such as endocytosis or intercellular membrane transfer (trogocytosis) can also facilitate antigen escape, allowing tumour cells to dynamically hide from CAR‐T cells [109, 110]. To address disease relapse caused by this dynamic immune escape, multiple strategies have been developed. First, dual‐ and multi‐target CAR‐T cells are designed to mitigate antigen escape risk. By broadening antigen recognition to target multiple TAAs or TSAs simultaneously, these cells can more effectively counter tumour heterogeneity and antigen downregulation [111, 112]. Second, combination strategies that promote endogenous immune activation, such as CAR‐T‐induced ES, hold significant promise. By expanding the immune response to new antigens presented by dying tumour cells, these approaches can overcome antigen escape and provide a more durable effect [113]. With the continued advancement of these strategies, CAR‐T cell therapy is expected to more effectively adapt to and address the dynamic challenge of immune escape, thereby improving therapeutic success rates and patient survival outcomes.

#### 3.3.1 Dual‐Target CAR‐T Cells to Overcome Single‐Antigen Escape

Dual‐target CAR‐T cells simultaneously recognize and target two distinct antigens, reducing immune escape and addressing lineage switching, which is commonly observed during tumour progression [7, 19]. This strategy enhances antigen recognition specificity and reduces treatment failure caused by the loss of a single antigen. Furthermore, the incorporation of logic‐gated circuits into CAR‐T cells enables more flexible and controllable dual‐targeting strategies. By incorporating synthetic “switch” mechanisms, engineered T cells are activated under precise antigen‐recognition conditions, enhancing targeting precision and therapeutic safety while mitigating antigen escape linked to single‐target loss [114].

Sequential therapy, a classic dual‐target CAR‐T strategy, involves infusing CAR‐T cells targeting different antigens either sequentially or concurrently to improve therapeutic efficacy and reduce relapse due to antigen escape. In a phase II clinical trial (ChiCTR2000032211) investigating combined anti‐CD19 and anti‐CD22 CAR‐T therapy for paediatric B‐ALL, 99% of the 194 patients with relapsed or refractory disease achieved CR, with all patients becoming (MRD)‐negative, and the 12‐month event‐free survival (EFS) rate reached 73.5% [115]. These findings suggest that dual‐target CAR‐T therapy substantially improves both treatment durability and efficacy, particularly in mitigating antigen escape. Similarly, in a clinical trial combining anti‐CD19 and anti‐CD20 CAR‐T cells for R/R Diffuse Large B‐Cell Lymphoma (DLBCL) (NCT03207178), sequential administration resulted in an encouraging objective response rate (ORR) of 81.0%, a CR rate of 52.4%, and a median progression‐free survival (mPFS) of 5.0 months, indicating combined treatment of multiple targets effectively reduces the problem of antigen loss commonly seen in single‐target therapy [111]. Moreover, a trial combining anti‐BCMA and anti‐CD19 CAR‐T cells in R/R MM (ChiCTR‐OIC‐17011272) reported an ORR of 92% and a CR rate of 60%. Among 56 evaluable patients, 77% achieved MRD negativity, and the mPFS reached 18.3 months. These clinical findings demonstrate that dual‐target therapy significantly prolongs therapeutic effects and mitigates PD due to antigen escape [112].

Beyond the classical sequential therapy, dual‐target CAR‐T therapy has evolved into multiple strategic formats, including co‐transduction, parallel bivalent and tandem/looped bivalent CAR designs (Figure 4 and Table 1). Among these strategies, the integration of synthetic logic‐gated circuits into CAR‐T systems – particularly through the incorporation of switch mechanisms – has markedly improved antigen specificity and safety profiles [114]. The “OR” logic gate‐based dual‐target CAR‐T design is a prominent strategy aimed at broadening target coverage and overcoming antigen escape, enabling CAR‐T cells to be activated upon recognition of either one of the targeted TAAs [116]. In the dual‐independent CAR format, one of the “OR” logic gate‐based dual‐target CAR‐T designs, two distinct CAR constructs targeting separate antigens are co‐expressed within a single T cell. Activation of either CAR is sufficient to trigger T cell effector functions. For instance, GD2/B7‐H3 dual CAR‐T cells demonstrated potent antitumour efficacy in neuroblastoma in vivo models. Compared to designs integrating multiple co‐stimulatory domains (e.g., CD28 and 4‐1BB) within a single CAR molecule, the split co‐stimulatory architecture showed enhanced T cell activation and effectively limited immune escape driven by antigen heterogeneity [117]. Moreover, emerging studies have explored synergizing TCR and CAR signalling pathways through engineered cross‐talk. This signal optimization enhances antitumour activity while preserving target specificity and minimizing off‐tumour effects [118].

**FIGURE 4:**
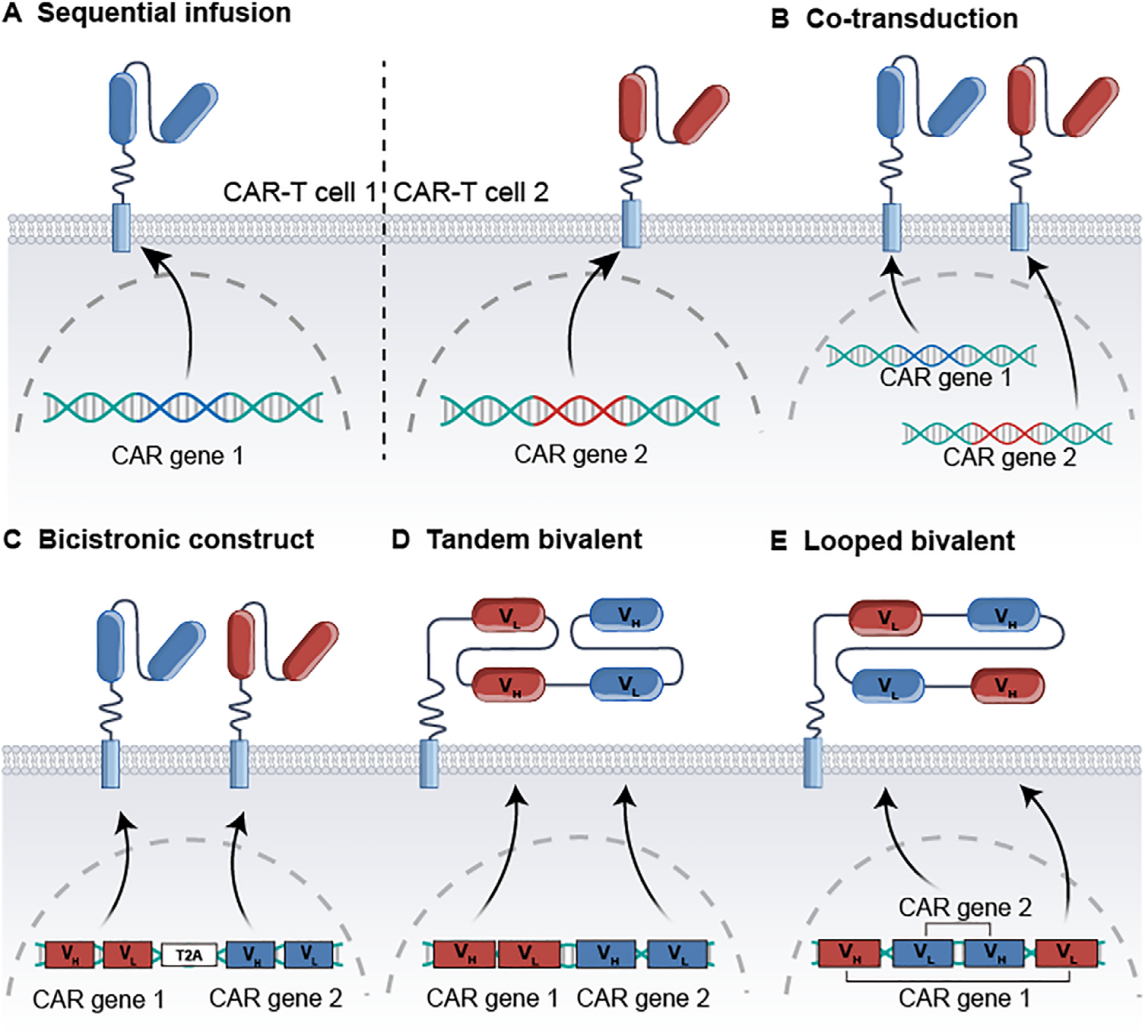
Schematic illustration of four dual‐target CAR‐T cell design strategies. (A) Sequential infusion: Two single‐target CAR‐T cell products, each targeting a different antigen, are infused at separate time points to achieve multi‐antigen targeting. (B) Co‐transduction: Two distinct CAR constructs are delivered into the same T cell population using separate vectors, resulting in a mixed population of cells expressing either one or both CARs. (C) Bicistronic construct (parallel bivalent CARs): A single vector expresses two full‐length CARs connected by an IRES or 2A peptide, enabling the same T cell to independently recognize two antigens. (D) and (E) Tandem and looped bivalent CAR: Two scFvs targeting different antigens are linked in tandem (or in a loop configuration) within a single CAR molecule, sharing the same transmembrane and signalling domains to allow multi‐antigen recognition through a unified structure.

**TABLE 1:** Comparison of different dual‐target CAR‐T strategies.

Strategy	Advantages	Disadvantages	Ref.
**Sequential infusion**	Requires minimal modification; enables combination of two single‐target CAR constructs; allows for adjusted dosing of each CAR product	High manufacturing cost; necessitates coordination and timing for two separate infusions of distinct products	[119]
**Co‐transduction**	Offers flexible control over the CAR ratio; avoids limitations of single‐vector designs; reduces risk of vector overload	Potential for uneven expression levels; low proportion of dual‐CAR^+^ cells; risk of competitive exhaustion; complex process	[120]
**Bicistronic construct (parallel bivalent CARs)**	Simplifies manufacturing; ensures expression of both CARs in the same T cell; allows for controllable expression levels	Requires complex vector design; may lead to imbalanced CAR expression	[121]
**Tandem / looped bivalent CAR**	Compact design; enables cooperative dual‐antigen recognition; simple to produce (two scFvs linked in one CAR using a single transmembrane and signalling domain)	Risk of conformational interference; limited recognition efficiency; challenging to tune affinity	[122]

#### 3.3.2 Epitope Spreading Induced by CAR‐T Combination Therapies

ES was first identified in autoimmune disease models [123]. It describes a process in which the immune response, initially directed against a specific epitope of an antigen, gradually expands to recognize other epitopes within the same antigen molecule (intramolecular spreading) or from other related antigen molecules (intermolecular spreading) [124]. In cancer immunotherapy, ES is considered a crucial endogenous mechanism that enhances immune response breadth, thus contributing to the control of antigen heterogeneity and escape mechanisms [125]. In CAR‐T cell therapy, the specific cytotoxic effect mediated by CARs leads to tumour cell lysis and death, releasing large amounts of intracellular proteins, apoptotic bodies, and potential neoantigens. These tumour‐derived components are captured and processed by antigen‐presenting cells (APCs), such as dendritic cells (DCs), and presented through both MHC class I and II pathways to CD8^+^ and CD4^+^ T cells. This process activates endogenous T cell populations to form a polyclonal immune response, capable of recognizing and attacking other tumour antigens not originally targeted by CAR‐T cells, amplifying the breadth and durability of the immune effect [126, 127, 128].

Lai et al. reported a novel cell therapy strategy in which T cells were engineered to co‐express Flt3 ligand (Flt3L), a cytokine critical for dendritic cell development, enhancing dendritic cell expansion and activation. This promoted antigen cross‐presentation and the occurrence of ES. Their study demonstrated that treatment with Flt3L‐expressing T cells not only exerted direct cytotoxicity against tumour cells but also stimulated endogenous T cell responses specific to non‐targeted tumour antigens, inducing broad‐spectrum antitumour immunity [113]. Similarly, Leyuan Ma et al. described an in vivo vaccine approach in which CAR ligands were synthetically conjugated to amphiphilic polymer–lipid tails. This strategy was shown to drive ES and engage the endogenous immune system, helping overcome immune escape by antigen‐negative tumour cells [129].

Such findings highlight the potential of ES as a self‐amplifying mechanism that extends the antigenic coverage of CAR‐T therapy and recruits the host's endogenous immune system into the antitumour response. Rather than relying solely on the direct cytotoxicity of CAR‐T cells, leveraging ES may represent a pivotal shift toward systemic and durable tumour control. Future strategies should aim to harness and refine this process – through rational combination therapies or CAR designs that promote antigen cross‐presentation – to mitigate antigen escape and enhance long‐term treatment outcomes.

### 3.4 Dynamic Optimization of Accessibility: From Autologous to “Off‐the‐Shelf” CAR‐T

Currently approved CAR‐T products primarily rely on lentiviral or γ‐retroviral transduction technologies to integrate and express CAR genes. However, this manufacturing approach entails significant limitations, including insufficient T cell expansion in vitro, inconsistent viral transduction efficiency, potential risks of insertional mutagenesis, and high production costs, all of which restrict the broad clinical adoption of CAR‐T therapy (Figure 5 and Table 2) [130]. Consequently, researchers are actively developing alternative platforms with broader applicability, improved manufacturing efficiency, and lower costs. Among these, two prominent approaches – ex vivo‐engineered universal CAR‐T (UCAR‐T) cells and in vivo CAR‐T cell induction – have shown considerable potential in pre‐clinical studies to reduce production costs while preserving therapeutic efficacy.

**FIGURE 5:**
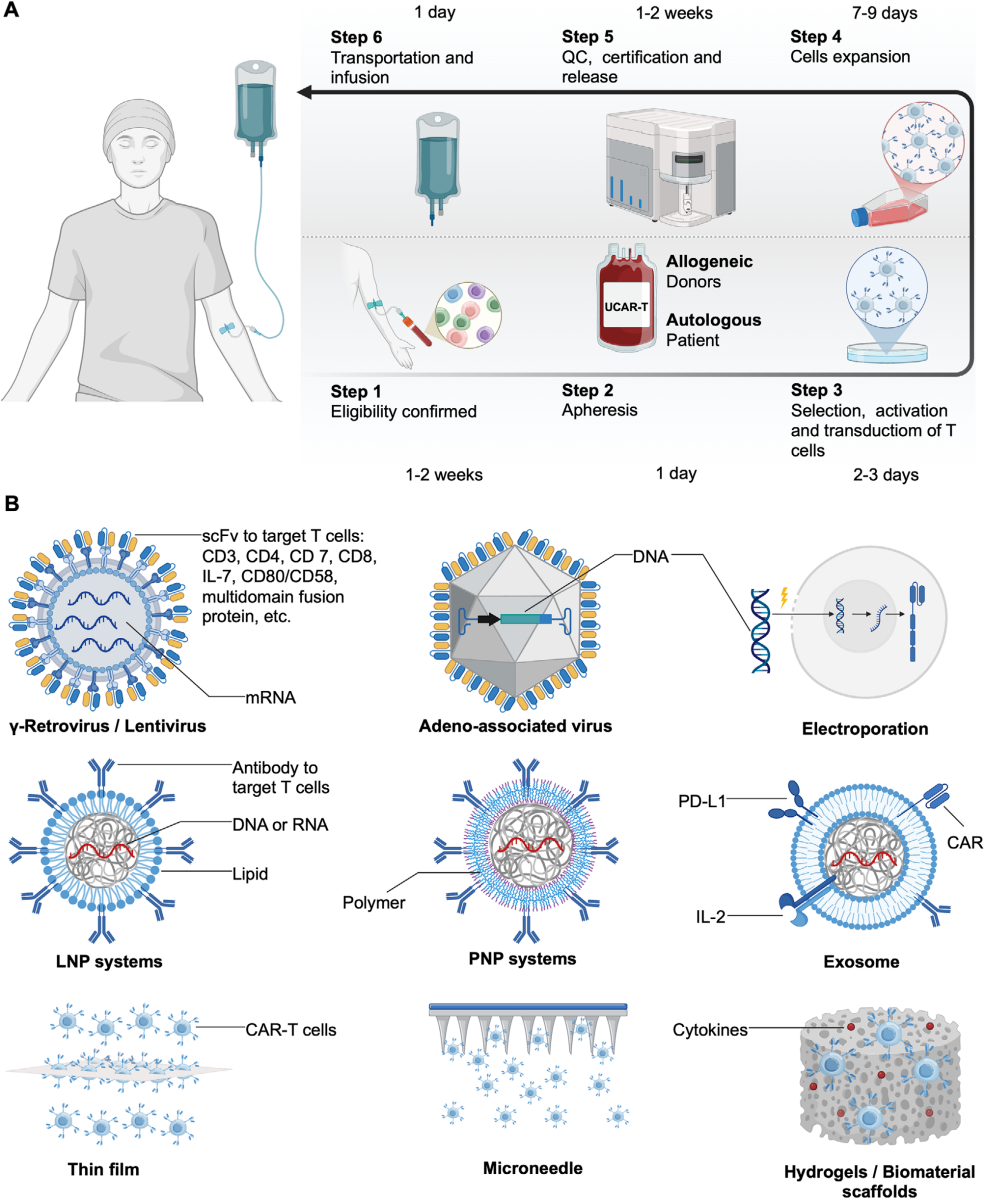
Comparative overview of ex vivo and in vivo CAR‐T cell manufacturing strategies. (A) Workflows for ex vivo autologous and allogeneic CAR‐T manufacture (UCAR‐T). For autologous manufacture, patient eligibility is confirmed; for allogeneic manufacture, donor eligibility is assessed. Autologous products use patients’ peripheral blood mononuclear cells (PBMCs) collected by apheresis, whereas allogeneic products derive from donor PBMCs, umbilical‐cord blood (UCB), or induced pluripotent stem cells (iPSCs). T cells are subsequently selected, activated, and genetically modified. Then cells are expanded in culture, undergo quality‐control testing, and are then released. Finally, the product is transported and infused. Personalized CAR‐T manufacturing still faces challenges such as high costs and limited accessibility. The end‐to‐end cycle requires 3–6 weeks and is associated with substantial lot‐to‐lot variability. Moreover, manufacturing can fail if patients provide insufficient or dysfunctional T cells, and PD during the wait may close the therapeutic window. Pre‐manufactured allogeneic CAR‐T products circumvent these delays by providing off‐the‐shelf therapy. (B) Delivery strategies for in vivo CAR‐T therapy. Systemic in vivo gene‐delivery platforms and local CAR‐T cell depots. Systemic in vivo approaches deliver CAR genetic cargo directly to circulating T cells, bypassing ex vivo expansion. Lentiviral and γ‐retroviral vectors integrate CAR transgenes into T cell genomes, enabling durable expression. Adeno‐associated viruses (AAVs) mediate episomal expression or, when paired with transposon or CRISPR/Cas9 systems, stable genomic insertion. Non‐viral carriers – lipid nanoparticles (LNPs), polymeric nanoparticles (PNPs), and exosomes – deliver CAR‐encoding mRNA or plasmid DNA. mRNA is translated directly in the cytoplasm, whereas plasmid DNA requires nuclear entry for transcription. Additionally, Antibody‐conjugated LNPs further enhance T cell targeting. Electroporation transiently permeabilizes cell membranes, permitting uptake of mRNA, plasmid DNA or CRISPR/Cas9 ribonucleoproteins for temporary CAR expression or genome editing. Advanced carriers can co‐encapsulate CAR cargo with immunomodulators (e.g., IL‐15 mRNA) for combinatorial control. Local‐delivery devices include thin films, microneedle patches, and injectable hydrogels or biomaterial scaffolds. Thin‐film systems immobilize CAR‐T cells on micro‐structured nitinol surfaces. After implantation, they permit sustained release and enhance CAR‐T cell retention. This promotes prolonged retention and localized activity of CAR‐T cells at the tumour site. Microneedle patches penetrate tumour tissue, distributing CAR‐T cells directionally within the lesion. Injectable hydrogels or biomaterial scaffolds tether or encapsulate CAR‐T cells within the TME, enabling controlled release and microenvironmental adaptation. This figure is created by BioRender (https://app.biorender.com).

**TABLE 2:** Overview of delivery methods and bioengineering approaches for CAR‐T cell production.

Delivery method		Delivered cargo	CAR DNA integration mechanism	Advantages	Limitations
**Viral delivery systems**					
γ‐Retrovirus/Lentivirus		RNA	Self‐integrating expression	High transduction efficiency; long‐term gene expression; suitable for hard‐to‐transfect cells	Risk of insertional mutagenesis; high immunogenicity and tumourigenic potential
AAV		DNA	Transposon system; CRISPR/Cas9 system	Low immunogenicity, high safety, relatively stable gene expression	Lacks an enveloped membrane for targeted delivery; in vivo use demands precise serotype selection
**Non‐viral delivery systems**					
Electroporation		mRNA; DNA	Transposon system; CRISPR/Cas9 system	Simple, broadly applicable; suitable for T cells ex vivo or local in vivo use	Risk of membrane rupture and cytotoxicity; loss of cytoplasmic content may alter gene‐expression profiles
Nanosystems	Lipid‐based (LNPs)	CAR mRNA; CAR DNA	Transposon system; CRISPR/Cas9 system	Non‐viral; low immunogenicity; scalable; targetable; good biocompatibility; high bioavailability and flexible payloads	Potential hepatic and splenic toxicity; low transfection efficiency in lymphocytes
	Lipid‐based (exosomes)	mRNA; DNA; Proteins; siRNA	Transposon system; CRISPR/Cas9 system	Low immunogenicity; high biocompatibility; natural targeting capability; can penetrate solid tumour barriers	High production cost; complex engineering; source‐standardization challenges; early‐stage clinical development
	Polymer‐based	mRNA; DNA	Transposon system; CRISPR/Cas9 system	Easy to prepare; low cost; tuneable release profiles	Reduced stability and transfection efficiency; potential immune activation
Hydrogels/bioinstructive implantable scaffolds		CAR‐T cells	Transposon system; CRISPR/Cas9 system	Enables local controlled release; reduces systemic toxicity	Restricted application scope; early‐stage clinical development
Films (local sustained release carriers)		CAR‐T cells	Transposon system; CRISPR/Cas9 system	Sustained and stable local delivery; suitable for tumour sites	Restricted to local use; low cellular loading capacity
Microneedles (subcutaneous delivery)		CAR‐T cells	Transposon system; CRISPR/Cas9 system	Easy to use; suitable for local administration of superficial tumours	Limited payload and penetration; unsuitable for deep tissues

Abbreviation: siRNA, small interfering RNA.

#### 3.4.1 Ex Vivo Preparation of CAR‐T Cells: Batch Production and Allogeneic Cell Sources

Allogeneic CAR‐T cells, utilizing T cells from healthy donor peripheral blood, umbilical cord blood (UCB), or induced pluripotent stem cells (iPSCs), offer the potential for standardized, batch production and off‐the‐shelf availability, thereby markedly reducing the per‐unit treatment cost and shortening patient waiting times (Figure 5A) [131]. A single apheresis sample from a healthy donor can yield sufficient material to produce multiple therapeutic doses of allogeneic CAR‐T cells. Cryopreserved banks permit repeat dosing and provide contingency stock in the event of manufacturing failure [132]. Furthermore, iPSC‐derived T cells can be expanded into HLA‐matched CAR‐T banks; targeted TCR deletion further mitigates GvHD [132, 133, 134]. Hosking et al. generated iPSCs‐derived allogeneic CAR‐T cells. By incorporating a high‐affinity HER2 CAR, a CD16a Fc receptor, and functional modules such as IL‐7R, TGF‐β/IL‐18R fusion, and CXCR2, they effectively enhanced specific targeting of HER2‐positive breast cancer, improved tumour infiltration, and prolonged cellular persistence, while demonstrating low toxicity to normal HER2‐expressing tissues [135]. UCB is likewise advantageous because naive T cells confer intrinsic immune tolerance. Consequently, incomplete HLA matching is acceptable, broadening donor‐recipient compatibility and reducing immunological complications [136, 137, 138]. Viral vectors, Sleeping Beauty transposons and CRISPR/Cas9 further streamline CAR construction, lowering cost while improving genomic stability [139, 140, 141, 142, 143]. Collectively, these advances herald truly scalable, off‐the‐shelf CAR‐T products. Nonetheless, hurdles – including host rejection, limited persistence and on‐target toxicities – remain. Ultimate success hinges on marrying precise immune engineering with rigorous clinical validation to ensure that convenience never compromises efficacy or safety.

#### 3.4.2 In Vivo Engineering of CAR‐T Cells: Breaking Through Cell Manipulation Bottlenecks and Enabling Enhanced Treatment

In contrast to conventional ex vivo manufacture, in vivo CAR‐T generation bypasses cell harvest, culture expansion, and reinfusion, offering a simplified, immediate therapeutic strategy that significantly reduces production cost and turnaround time (Figure 5B) [144]. This approach delivers CAR‐encoding cargo directly to circulating T cells, which subsequently express the receptor in situ, eliminating the need for GMP‐grade cell manipulation, cold‐chain logistics, and individualized quality control [145]. Among emerging delivery platforms, nanoparticles (NPs) have gained increasing attention for their potential to facilitate efficient in vivo CAR‐T programming [146]. Lipid nanoparticles (LNPs) and polymeric nanoparticles (PNPs), in particular, offer not only high biocompatibility but also modularity and tunability, enabling programmable delivery with enhanced precision and functional complexity [147].

Nanoparticle platforms – including LNPs, PNPs and extracellular vesicles (EVs) – are rapidly gaining prominence in preclinical research and hold considerable promise for overcoming the limitations of ex vivo CAR‐T manufacturing, such as autologous cell harvest, expansion, and reinfusion steps [148, 149, 150]. Recently, Bimbo et al. developed a novel dual‐targeted lipid nanoparticle (tLNP) system capable of simultaneously recognizing CD7 and CD3 on T cells [151]. Under CD3/CD28 preactivation, this platform achieved nearly 50% minicircle DNA (mcDNA) transfection efficiency and induced over 60% of T cells to express the activation marker CD25. Because LNPs avoid genomic integration, they confer superior safety and reversibility [152, 153]. Moreover, multiple studies have demonstrated that CAR mRNA encapsulated in LNPs can enable highly efficient, controllable, and reproducible in vivo generation of CAR‐T cells across various animal models [154, 155, 156]. LNP‐based CAR delivery technologies have recently been extended beyond T cells to other immune subsets – for instance, enabling efficient reprogramming of M2‐polarized macrophages to express CARs targeting fibroblast activation protein‐α (FAP) [157]. These advances highlight that NPs are not merely passive delivery vehicles but can play an active and therapeutic role in overcoming barriers that limit CAR‐T efficacy, particularly within the hostile milieu of solid tumours.

Notably, nanotechnology not only facilitates in vivo CAR‐T cell generation but also enhances CAR‐T functionality across multiple dimensions. A growing number of functional nanoparticle platforms have been engineered to promote CAR‐T cell expansion, infiltration, and sustained functional persistence within the TME, which has been proposed as “CAR‐T Nanosymbionts” [158, 159]. To enhance CAR‐T cell expansion and systemic immune activation, BioNTech has developed an RNA vaccine encoding the CLDN6 antigen, which induces CLDN6 expression in APCs and thereby stimulates the controlled expression of previously infused CLDN6‐targeted CAR‐T cells in vivo in patients with relapsed/refractory CLDN6‐positive solid tumours [160]. NPs can also function as active TME modulators to facilitate CAR‐T cell infiltration and sustain functional persistence. For example, LNPs co‐encapsulating a STING agonist and a Toll‐like receptor 4 (TLR4) ligand, combined with senescence‐inducing RAS‐targeted therapy, have been shown to remodel the TME in pancreatic ductal adenocarcinoma, leading to sustained CD8^+^ T cell activation and antitumour responses [161]. Similarly, polymeric micelle NPs delivering a TLR7/8 agonist and a PI3Kδ inhibitor reprogrammed the TME by promoting M1 macrophage polarization and selectively suppressing immunosuppressive MDSCs and Tregs [162]. In addition, multiple mRNA vaccine strategies have been employed to generate CAR‐T cells in vivo and overcome barriers in solid tumours. These include synthetic VHH‐encoding mRNA for converting “cold” or antigen‐low tumours into immunologically visible targets, as well as antigen‐spreading strategies to address heterogeneity and antigen loss [129, 163]. Integration of prostate‐specific membrane antigen (PSMA)‐targeted ligands and imaging probes into LNP‐mRNA‐CAR systems enables real‐time, in situ monitoring of CAR‐T cell distribution and activity, providing feedback for dose adjustment, temporal intervention, and therapeutic efficacy evaluation [164]. Moreover, through localized actions such as remodelling the extracellular matrix, modulating stromal cell activity, or targeting immunosuppressive myeloid cells, NPs can establish a transient but critical “window of opportunity” for CAR‐T infiltration, persistence, and cytotoxic function [165]. Despite current challenges – such as biosafety concerns, suboptimal transfection efficiency, and limited subset‐specific targeting – nanoparticle platforms, once clinically validated, may hold immense promise due to their modularity, programmable architecture, and precise spatiotemporal control, potentially broadening the therapeutic landscape of CAR‐T cells in solid tumours and other complex immune‐mediated diseases [166].

### 3.5 Dynamic Control of Toxicity: The Final Frontier for CAR‐T Therapy

Despite the remarkable potential of CAR‐T cell therapy in combating solid tumours, its safety profile has become an increasing concern, owing to the distinct toxic phenotypes, underlying mechanisms, and management strategies that differ substantially from conventional treatments [167]. Across all tumour types, CAR‐T therapy is associated with varying degrees of treatment‐related toxicities [168].

In current clinical studies, CRS and ICANS remain the most commonly observed AEs [169]. CRS typically presents with fever, hypotension, and hypoxia, driven by the exuberant release of inflammatory cytokines – including IL‐6 and IFN‐γ – after CAR‐T cell activation [170]. The onset of CRS varies significantly, generally occurring 2–7 days after infusion but may appear within hours under high‐dose regimens [171]. ICANS is mostly attributed to increased blood–brain barrier permeability and neuroinflammatory responses, presenting as altered consciousness, aphasia, or even seizure‐like episodes [172]. It can occur concurrently with CRS or, more commonly, after CRS resolution – typically 3–5 days post‐infusion, though delayed onset beyond 3 weeks has also been reported [173]. In addition, immune effector cell‐associated haematotoxicity (ICAHT) – including neutropenia, anaemia, and thrombocytopenia – is frequent, especially when CAR‐T therapy follows chemo‐ or radiotherapy [174].

Relative to haematological malignancies, CAR‐T therapy for solid tumours is more susceptible to OTOT toxicity, wherein low‐level antigen expression on healthy tissues triggers collateral damage [31, 175]. Several clinical trials have reported typical OTOT events during CAR‐T treatment of solid tumours. For example, CAR‐T cells targeting carbonic anhydrase IX (CAIX) in clear cell carcinoma caused cholangitis‐associated hepatotoxicity; CEACAM5‐targeted CAR‐T therapy led to severe pulmonary toxicity, likely due to low‐level antigen expression in healthy lung tissue, and a HER2 CAR‐T infusion precipitated fatal acute respiratory failure [31, 40, 176].

To mitigate these multifaceted toxicities, investigators have advanced a multi‐layered framework of safety controls and interventions. Systemic toxicities are managed with established algorithms, including staged administration of the IL‐6 receptor antagonist Tocilizumab and corticosteroids, which quell cytokine storm and neurotoxicity [177]. Meanwhile, efforts at upstream prevention have focused on optimizing treatment safety from two key aspects: target selection and CAR construct design. On the one hand, selecting TSAs (e.g., EGFRvIII, CLDN18.2) can significantly reduce the risk of OTOT effects [178, 179]. On the other hand, the incorporation of controllable CAR design elements – such as logic‐gated systems (e.g., AND gate), inducible safety switches (e.g., iCasp9), and local delivery approaches – enhances the precision and controllability of CAR‐T cells [180, 181, 182, 183]. In addition, the combination of optimized targeting strategies with drug co‐administration has demonstrated favourable safety profiles in multiple clinical studies. For example, EGFRvIII CAR‐T cells plus pembrolizumab elicited no DLTs or CNS/ICANS events in a phase I GBM trial (NCT03726515) [178]. Likewise, mesothelin CAR‐T cells with Pembrolizumab showed encouraging safety in malignant pleural disease (phase I, NCT02414269) [184]. Moreover, in an open‐label study (NCT05660369), three patients with recurrent GBM received TEAM‐engineered CAR‐T cells secreting bispecific antibodies against EGFRvIII and wild‐type EGFR, with no grade ≥3 AEs or DLTs [185]. Ultimately, CAR‐T safety in solid tumours depends not merely on toxicity management but on embedding precision and control into the therapeutic design. Mitigating OTOT injury and immune‐mediated damage therefore demands a shift from reactive management to proactive, antigen‐specific, modular safety engineering.

## 4 Dynamic Immune Reprogramming: Extending Applications From Oncology to Autoimmune Diseases

### 4.1 Haematologic Malignancies

Haematologic malignancies, including leukaemia, lymphoma, and MM, were the first disease types in which CAR‐T cell therapy achieved clinical success. Among them, ALL remains a leading cause of cancer‐related death in individuals younger than 20 years worldwide. In 2021, an estimated 53,485 paediatric and adolescent ALL cases and 23,991 related deaths were recorded globally [186, 187]. Lymphoma is likewise prevalent, with 537,904 Hodgkin lymphoma (HL) and 1,741,300 non‐Hodgkin lymphoma (NHL) cases reported worldwide in 2019 [188]. MM primarily affects older adults and remains incurable owing to frequent relapse and drug resistance. In 2022, the global burden of MM was 188,000 new cases and 121,000 deaths [189, 190, 191]. Despite continuous optimization of current treatment strategies, many patients relapse or develop resistance after multiple treatment lines, resulting in a poor prognosis [192, 193, 194]. To date, 14 CAR‐T cell products have received regulatory approval worldwide (Table 3), all for haematological indications. After years of research and clinical exploration, CAR‐T therapy has achieved significant advances in treating haematologic cancers, with the development of novel targets steadily progressing (Table 4). Currently, CAR‐T cell therapy is now integrated into third‐line and later regimens for various R/R haematologic malignancies, providing new options and significantly extending long‐term survival for selected patients [195].

**TABLE 3:** Global commercialized CAR‐T products.

Trade name (Generic name)	1st approval date and country	Company	Target	Approved indications	Key clinical outcomes	Ref.
Kymriah (Tisagenlecleucel, Tisa‐cel)	2017.8, US	Novartis	CD19	R/R B‐ALL ≤25 years old^a^; Adult R/R LBCL after ≥2 prior lines (incl. DLBCL NOS, HGBL, tFL); Adult R/R FL after ≥2 prior lines	ORR: 82% mEFS: 24 months OS (3 years): 63% AEs (>1year): 29% Grade 3/4	[196]
Yescarta (Axicabtagene ciloleucel. Axi‐Cel)	2017.10, US	Gilead Sciences	CD19	Adult LBCL – 1L refractory or early relapse; Adult R/R LBCL after ≥2 prior lines; Adult R/R FL after ≥2 prior lines^a^	ORR: 90% mPFS: 62.2 months mFU: 64.6 months	[197]
	2021.6, CN	FosunKite		R/R large B‐cell lymphoma after ≥2 prior lines (incl. DLBCL NOS, PMBCL, HGBL, and tFL)^a^	OS: (4 years): 54.6% mPFS: 14.7 months AEs: 100%	[198]
Tecartus (Brexucabtagene autoleucel, brexu‐cel)	2020.7, US	Gilead Sciences	CD19	Adult R/R MCL; Adult R/R B‐ALL^a^	CR/CRi: 80% CRS (100 days): 81% any Grade NT/ICANS (100 days): 46% any Grade	[2]
Breyanzi (Lisocabtagene maraleucel)	2021.2, US	Bristol Myers Squibb	CD19	Adult R/R LBCL; Adult R/R CLL/SLL after ≥2 prior lines (incl. BTKi & BCL‐2i)^a^; Adult R/R FL after ≥2 prior lines; Adult R/R MCL after ≥2 prior lines (incl. BTKi)	CR/CRi: 20% mPFS: 11.9 months mOS: 30.3 months CRS: 85% any Grade NE (neurologic events): 45% any Grade	[199]
Abecma (Idecabtagene vicleucel, Ide‐Cel)	2021.3, US	Bristol Myers Squibb and Bluebrid Bio	BCMA	Adult R/R MM after ≥2 prior lines (incl. IMiD, PI, anti‐CD38)^a^	ORR: 71% mPFS: 13.3 months NT/ICANS: 15% CRS: 88% AEs: 93% Grade 3/4	[200]
Beinuoda (Relmacabtagene Autoleucel, Relma‐Cel)	2021.9, CN	JW Therapeutics	CD19	Adult R/R LBCL after ≥2 prior lines^a^; Adult R/R FL (POD24) after ≥2 prior lines	ORR (3 months): 58.6% CR: 51.7% PFS (6 months): 54.2% OS (6 months): 90.8% CRS: >5% Grade ≥3 Lab abnormalities: >10% ≥3 Grade	[201]
Carvykti (Ciltacabtagene Autoleucel, Cilta‐Cel)	2022.2, US	Legend Biotech	BCMA	Adult R/R MM after ≥1 prior line (incl. PI, IMiD, lenalidomide‐refractory)^a^	ORR (12 months): 84.6% PFS: 75.9% CRS: 76.1% NT/ICANS: 4.5% Grade 1/2	[202]
	2024.8, CN				ORR: 87.9% CR/sCR: 79.3% mPFS: 30.13 months mFU: 37.29 months CRS: 97.9%, 35.4% Grade 3/4	[203]
Fukesu (Equecabtagene Autoleucel, Eque‐Cel)	2023.6, CN	IASO Bio & Innovent Biologics, Inc.	BCMA	Adult R/R MM after ≥3 prior lines (incl. PI & IMiD)^a^	ORR (13.8 months): 96.0% CR (13.8 months): 74.3% PFS (12 months): 78.8% CRS: 93.2% NT/ICANS: 1.9% Grade 1/2	[204]
Yuanruida (Inaticabtagene Autoleucel, Inati‐Cel)	2023.11, CN	Juventas Cell Therapy Ltd.	CD19	Adult R/R B‐ALL^a^	ORR (3 months): 66.7% CRS: 8.3% NT/ICANS: 8.3% Grade ≥3	[205]
Saikaize (Zevorcabtagene Autoleucel, Zevor‐Cel)	2024.3, CN	CARsgen Therapeutics	BCMA	Adult R/R MM after ≥3 prior lines (incl. PI & IMiD)^a^	ORR (PR or better, 227 days): 92.8% CR/sCR: 42.2% PFS (6 months): 90.2% CRS: 90.2% NT/ICANS: 2% Grade 1	[206]
Aucatzyl (Obecabtagene Autoleucel, Obe‐Cel)	2024.11, US	Autolus Therapeutics	CD19	Adult R/R B‐ALL^a^	ORR: 77% CR: 55% mOS: 15.6 months mFU: 20.3 months CRS: 2.4% Grade ≥3 NT/ICANS: 7.1% Grade ≥3	[207]
HICARA	2025.7.29, CN	Hrain Biotechnology Co., Ltd.	CD19	R/R NHL^a^; Adult R/R LBCL after ≥2 prior lines	Best ORR: 74.1% Best CRR: 49.4% mDOR: 339 days mPFS: 176 days CRS: 95.1%, 3.7% Grade ≥3 ICANS: 8.6%	[208]

^a^ The primary clinical data for this indication are summarized in the “Key clinical outcomes” column.

Abbreviations: BCL‐2i, B‐cell lymphoma‐2 inhibitor; BTKi, Bruton's tyrosine kinase inhibitor; CLL, chronic lymphocytic leukaemia; Cri, complete response with incomplete haematologic recovery; FL, follicular lymphoma; ICANS, immune effector cell‐associated neurotoxicity syndrome; IMiD, immunomodulatory drug; MCL, mantle cell lymphoma; mEFS, median event‐free survival; mFU, median follow‐up; mOS, median overall survival; NE, neurotoxicity events; NT, neurotoxicity; OS, overall survival; PI, proteasome inhibitor; POD24, progression of disease within 24 months; PFS, progression‐free survival; PR, partial response; sCR, stringent complete response; SLL, small lymphocytic lymphoma. Country codes: US (United States FDA), CN (China NMPA).

**TABLE 4:** Recent advances in promising clinical therapeutic targets for haematologic malignancies.

Tumour type	Target	Pts. number	Efficacy	Safety	Phase	Registration	Ref.
**Acute lymphoblastic leukaemia (ALL)**							
R/R T‐ALL	CD5	19	ORR/CR: 100% (by day 30) mFU: 14.3 months	DLT (21‐day): None CRS (≤ 30 days): 75%, 8 Grade 1 (fever), 4 Grade 2 (fever + hypoxia) NT/ICANS: 25%, 3 Grade 1 (headache, mild inattention), 1 Grade 2 (moderate inattention, cognitive difficulty) SAEs (≤30 days): 3 Grade 3 Klebsiella infections (Cohort B) Cytopenia: 100% Grade ≥3 GvHD (*n* = 11): 69% (skin/intestinal) Infections (late): 5 Grade 5	I	NCT05032599	[209]
R/R T‐ALL	CD7	17	ORR/CR: 94.1% mDOR: Ongoing, 11 relapse‐free at mFU of 15 months	CRS: 10 Grade 1, 3 Grade 2, No Grade ≥3 events ICANS: 2 Grade 1 (hand tremors, hyperesthesia), None Grade ≥2 ICAHT: 100% Grade ≥2 CTCAE: 29.4% Grade ≥3 infections (no ICU required)	I	NCT05043571	[210]
R/R B‐ALL	CD19‐CD20	194	ORR: 99.0% (95% CI: 97.5‐100) CR (MRD‐negative, *n* = 192); MRD: 100% Relapse: 43 (22.2% cumulative incidence); 24 CD19^+^/CD22^+^; 16 CD19^−^/CD22^+^; 1 CD19^−^/CD22^−^; 2 Unknown EFS (12‐month): 73.5% (95% CI: 67.3‐80.3) OS (12‐month): 87.7% (95% CI: 82.9‐92.9) mFU: 11.0 months (range 0.1‐32.4 months)	CRS: 88.0%, 28.4% Grade ≥3, 1 Fatal NT/ICANS: 20.9%, 4.0% Grade ≥3, 2 Fatal Seizures: 14.2% Grade 3–4, higher in pts. with CNS leukaemia Hypotension: 40.9% Grade 3–4	II	ChiCTR2000032211	[115]
R/R B‐ALL	CD22	21	ORR: 73.3% CR (CD19^−^/dim pts. *n* = 5): 100% mDOR: 6 months	CRS: 76.2%, 9 Grade 1, 7 Grade 2, 1 Grade 3 (DLT), 1 Grade 4 (DLT) NT/ICANS: None	I	NCT02315612	[211]
***Acute myeloid leukaemia (AML)***							
CD7^+^ R/R AML	CD7	10	CR: 70% mFU: 178 days (range 28–776) Relapse after CR (w/o HSCT): 3/4 (within 90 days) Ongoing remission: 1 (Day 401)	CRS: 80% (all mild) NT/ICANS: None Overall tolerability: Favourable	I	NCT04938115	[212]
R/R AML	CD33/Siglec‐3	4	CRi: 50% (Day 30)	CRS: 100%, 3 Grade 1–2, 1 Grade 4 DLT: 1 NT/ICANS: 50% Grade 1 GvHD: 50% Grade 1–2 Cytopenia: 100% Grade 2–4 Sepsis: 1	I	NCT04835519	[213]
Relapsed AML	CD38	6	CR/CRi (4‐week): 66.7% mCR/CRi duration: 191 days (range 117–261) Relapse rate (6‐month): 50% mOS: 7.9 months mLFS: 6.4 months	Toxicity: All AEs manageable Off‐target toxicity: None	I/II	NCT04351022	[214]
R/R AML	CD123	6	ORR: 50% SD/PD: 3 mOS: 160 days	CRS: 83%, 2 Grade 5 (deaths) NT/ICANS: None	I	NCT03766126	[215]
R/R AML	CLL‐1/CLEC12A	30	ORR: 73.3% CR/CRi: 40% MRD^−^, 33.3% MRD^+^	DLTs: None CRS: 100%; 12 Grade 3–4 NT/ICANS: 1 Grade 4 (manageable) Haematologic AEs: 100%, Grade 3–4 Cytopenia common	I	ChiCTR2000041054	[216]
R/R AML/MDS	NKG2DL	16	ORR (*n* = 12 evaluable): 25%	MTD: Not reached CRS: 31%, 1 Grade 3–4 (DLT) Treatment‐related deaths: None TRAEs: 44% Grade 3–4	I	NCT03018405	[217]
***Lymphoma, chronic lymphocytic leukaemia (CLL)***							
TCL	CD5	9	ORR: 44% PFS/OS: CAR T cells persisted ≥6.5–9 months in CR cases; 1 CR patient remained disease‐free 41 months post‐transplant	CRS or NT/ICANS: None Grade ≥3	I	NCT0308190	[218]
R/R PTCL	CD7	5	ORR: 80% CR: 60% mFU: 234 days (2 deaths post‐CR)	CRS: 100% Grade 1–2 NT/ICANS: None	I	NCT04928105	[219]
R/R LBCL	CD19	257	ORR: 73% CR: 53% mPFS: 6.8 months mOS: 27.3 months mFU: 19.9 months	CRS: 42% Grade ≥1, 2% Grade ≥3 NEs: 30% Grade ≥1, 10% Grade ≥3	I II/III	NCT02631044 NCT03435796	[220]
R/R B‐NHL	CD19‐CD20	47	ORR: 91.5% CR: 85.1% DOR (2‐year): 66.0% PFS: 62.6% OS: 76.5%	DLT: None CRS: 93.8% NT/ICANS: 6.3% SAE: 20.8%	I	NCT04655677 NCT04696432 NCT04693676	[221]
CAR19‐relapsed/CD19^−^ LBCL	CD22	38	ORR: 68% (95% CI 51–83) CR: 53% (36‐69) mDOR: 27.8 months mPFS: 3.0 months mOS: 14.1 months	CRS: 95%, 1 Grade 3 NT/ICANS: None Grade ≥3	I	NCT04088890	[222]
R/R HL/CD30^+^ T‐NHL	CD30	10	ORR: 100% CR: 60%	DLT: None CRS: 60% Grade 1 NT/ICANS: None	I	NCT04653649	[223]
R/R CD37^+^ lymphoid malignancies	CD37	5	ORR: 80% CR: 60%	Prolonged severe pancytopenia: 60% Cetuximab failed to deplete CAR in 2 pts. Haematopoiesis: restored via allo‐HSCT Severe non‐haematologic AEs: None	I	NCT04136275	[224]
R/R PTCL / CTCL	CD70	39	ORR: 46.2% (95% CI: 30.1–62.8) ORR (DL = 3 × 10^8^ cells, 9 × 10^8^ cells, *n* = 31): 51.6% (95% CI: 33.1–69.8) CR (DL = 3 × 10^8^ cells, 9 × 10^8^ cells, *n* = 31): 19.4% PR (DL = 3 × 10^8^ cells, 9 × 10^8^ cells, *n* = 31): 32.3%	CRS: 67%; 23 Grade 1–2, 2 Grade 3, 1 Grade 4 (DLT) NT/ICANS: 10% Grade 1–2 Grade 3–4 AEs: 36% Neutropenia, 28% Anaemia, 15% Thrombocytopenia SAEs: 64%, 36% Treatment‐related, 28% CRS (Most common) Deaths: 21 (16 PD)	I	NCT04502446	[225]
R/R TRBC1^+^ PTCL	TRBC1	10	ORR: 50% CMR: 30% PR: 20% mOS: 13.8 months	DLT: None CRS: 40% NT/ICANS: None	I/II	NCT03590574	[226]
CLL	ROR1	3	ORR: 67%	Well tolerated	I	NCT02706392	[227]
***Multiple myeloma (MM)***							
R/R MM	BCMA	117	ORR: 97% CR/sCR: 68% PFS (12 months): 79% (12 months); 66% (18 months) OS (12 months): 95% (12 months); 90% (18 months)	CRS: 85%; 80% Grade 1; 15% Grade 2; 1% Grade 5 ICANS: 8%; 3% Grade 1; 3% Grade 2; 1% Grade 3	II	NCT05396885	[228]
R/R MM	BCMA‐CD19	50	ORR: 92% sCR: 36%, CR: 24% MRD: 83% mPFS: 19.7 month mOS: 19.7 months	CRS: 92%, 8% Grade≥3 NT/ICANS: 4% Grade 1; no Grade ≥3 Haematologic AEs (Grade 3–4): 98% Neutropenia, 96% leukopenia, 64% anaemia, 66% thrombocytopenia Late AEs: 29% Prolonged cytopenia (>3 months); 90% B cell aplasia, 51% hypogammaglobulinaemia Mortality: 16%, 4 PD, 4 bleeding or infection	I/II	ChiCTR2000033567	[229]
R/R MM	BCMA‐CD38	23	ORR: 87% sCR: 52% MRD: 100% mPFS: 17.2 months	CRS: 87% NT/ICANS: None Haematologic AEs: Neutropenia (96%), leukopenia (87%), anaemia (43%), thrombocytopenia (61%)	I	ChiCTR1800018143	[230]
R/R MM	CS1 (SLAMF7)‐BCMA	16	ORR: 100% sCR: 46% MRD: 100% OS (1‐year): 72.73% PFS (1‐year): 56.26%	CRS: 38% NT/ICANS: None Haematologic: 100% Grade 3–4 leukopenia, 94% neutropenia, 100% lymphopenia, 13% anaemia, 31% thrombocytopenia Hepatotoxicity: 31% ↑ ALT/AST Immunotoxicity: 100% hypogammaglobulinaemia	I	NCT04662099	[231]
R/R MM/pPCL	BCMA‐NKG2A	5	ORR: 60% sCR: 40%, 1 R/R MM (sCR ongoing, mDOR >23 months), 1 pPCL (DOR = 20 months)	DLT / Death: None Haematologic AEs: 100% Grade 4 Cytopenia (↓lymphocytes, neutrophils, platelets) CRS: 40%, 1 Grade 1, 1 Grade 2 NT/ICANS & GvHD: None Infections: 1 Grade 3 pneumonia (resolved)	I	NCT05066022	[232]
R/R MM	GPRC5D	20	mFU: 10.71 months ORR: 100% (95% CI 83.2‐100) CR: 50%	DLT: None CRS: 60% Grade 1–2 ICANS: 5% Grade 3 TRAEs: 35% Treatment‐related deaths: None	I	NCT05838131	[233]

Abbreviations: AML, acute myeloid leukaemia; B‐NHL, B cell Non‐Hodgkin lymphoma; CMR, complete metabolic response; CTCAE, Common Terminology Criteria for Adverse Events; DL, dose level; DLT, dose‐limiting toxicity; DOR, duration of response; GI, gastrointestinal; GPRC5D, G Protein‐Coupled Receptor Class C Group 5 Member D; HSCT, haematopoietic stem cell transplantation; ICU, intensive care unit; LFS, leukaemia‐free survival; MDS, myelodysplastic syndrome; mDOR, median duration of response; mLFS, median leukaemia‐free survival; N/A, not available; NSCLC, non‐small cell lung cancer; pPCL, primary plasma cell leukaemia; PTCL, peripheral T cell lymphoma; Pts., patients; SAE, serious adverse event; SLAMF7/CS1, signalling lymphocytic activation molecule F7; TCL, T cell lymphoma; TNBC, triple‐negative breast cancer; TRBC1, T cell receptor beta constant 1.

*Note*: Some trials were referenced from secondary literature (reviews/meta‐analyses) without explicit NCT numbers.

#### 4.1.1 Leukaemia

CD19‐targeted CAR‐T cell therapy has demonstrated promising efficacy in B‐ALL [234]. However, many recipients ultimately experience PD – either “positive” relapse from limited CAR‐T cell persistence or “negative” relapse caused by antigen loss or down‐regulation [107, 235, 236, 237].

In patients with R/R B‐ALL, a phase II clinical trial involving 194 participants (ChiCTR2000032211) reported that coadministration of CD19 and CD20 CAR‐T cell therapy achieved an ORR of 99%, with a 100% rate of MRD‐negative CR. The 12‐month EFS and OS rates reached 73.5% and 87.7%, respectively. Despite impressive efficacy, CRS occurred in 88% and ICANS in 21% of patients [115]. CD22 is an additional B‐ALL target beyond CD19 and CD20. A phase I clinical study (NCT02315612) demonstrated an ORR of 84.6% and a CR rate of 73.3% among 21 patients with R/R B‐ALL, with a 100% CR rate observed in CD19‐neg or CD19‐dim patients. Although most patients experienced mild to moderate CRS, no ICANS occurred [211]. In T‐ALL, CD5 is a highly expressed surface antigen and a promising target. In a recent phase I clinical trial (NCT05032599), all 19 R/R T‐ALL patients attained CR within 30 days of CD5 CAR T infusion; mFU was 14.3 months. Grade 1–2 CRS occurred in 75% of patients, and mild ICANS was reported in 25%. Notably, GvHD and late infections were common after allogeneic products, underscoring the need for prolonged immune surveillance [209]. In AML, the lack of ideal TSAs has prompted evaluation of CD7, CD38, and CLL‐1 (CLEC12A). In a phase I clinical study among 10 patients with CD7^+^ R/R AML (NCT04938115), 70% achieved CR, with generally mild toxicity [212]. A CD38 CAR T trial (NCT04351022) yielded CR/CRi in 67% but a 6‐month relapse rate of 50%; toxicities were manageable [214]. A separate study on CLL‐1‐targeted CAR‐T therapy reported an ORR of 73.3%, with MRD‐negative CR/CRi achieved in 40% of patients, although all patients experienced grade ≥3 haematologic toxicities (ChiCTR2000041054) [216].

#### 4.1.2 Lymphoma

Currently, approved CD19‐directed CAR‐T therapies are primarily indicated for B cell non‐Hodgkin lymphoma (B‐NHL); however, some patients experience CD19 antigen escape (e.g., downregulation or loss of CD19 expression) or insufficient CAR‐T cell expansion and persistence, leading to PD [238]. These shortcomings underscore the imperative to optimize target selection and product architecture.

CD19 CAR‐T therapy is well validated in R/R LBCL. Two phase I trials (NCT02631044, NCT03435796; *n* = 257) produced an ORR of 73% and a CR of 53%. The mPFS and OS were 6.8 months and 27.3 months, respectively, with an overall manageable safety profile (Grade ≥3 CRS in 2% and Grade ≥3 neurotoxicity in 10%) [220]. Dual‐targeting CD19‐CD20 CAR‐T therapies have demonstrated enhanced efficacy in 47 efficacy‐evaluable patients with R/R B‐NHL, with an ORR of 91.5% and a CR rate of 85.1% (NCT04655677, NCT04696432, NCT04693676) [221]. Two‐year PFS and OS were 63% and 77%, with no dose‐limiting toxicities. These findings suggest that dual‐targeting strategies may help overcome antigen escape and prolong response durability. More recently, a first‐in‐class allogeneic γδ CAR‐T therapy targeting both CD19 and CD20 has entered clinical evaluation for R/R B‐NHL (NCT06395870) [239]. In this study, LUCAR‐G39D demonstrated favorable safety without dose‐limiting toxicities, neurotoxicity, or GvHD, while achieving an ORR of 70% with sustained responses and encouraging PFS, highlighting the potential of γδ CAR‐T platforms to overcome manufacturing constraints and antigen heterogeneity. Target discovery in T cell lymphomas is challenging, yet preliminary progress is emerging. CD7 CAR‐T therapy in five R/R PTCL patients achieved an 80% ORR and 60% CR (NCT04928105) [219]. Responses persisted over a mFU of 234 days, yet two patients died post‐CR, emphasizing the need for long‐term monitoring and combination strategies. Additionally, CD30 is also a promising target in classical HL and CD30‐positive T‐cell NHL. In NCT04653649, all ten patients responded (ORR = 100%; CR = 60%). Notably, treatment was limited to grade 1 CRS with no neurotoxicity or DLT, indicating a favourable safety profile and encouraging early efficacy [223].

#### 4.1.3 Multiple Myeloma

MM is the second most common haematological cancer. Despite advances in frontline therapy, most patients ultimately relapse or develop resistance [240, 241]. In recent years, BCMA‐targeted CAR‐T cell therapy has shown transformative activity in R/R MM [242, 243]. However, monospecific BCMA targeting can permit antigen escape and limit durability. Consequently, numerous trials are examining bi‐ or multi‐specific BCMA‐based CAR‐T constructs to improve efficacy and prolong remission.

In a CD19/BCMA dual‐target study (ChiCTR2000033567; *n* = 50 R/R MM), ORR was 92%, with sCR and CR of 36% and 24%. MRD negativity reached 83%, and mPFS and mOS were 19.7 months. CRS occurred in 92%, while the incidence of ICANS was 4% [229]. Another first‐in‐class autologous BCMA/CD19 dual‐target CAR‐T therapy has demonstrated encouraging activity in a phase 1b/2 study. Manufactured using the FasTCAR rapid platform to preserve naïve and central memory T‐cell phenotypes with strong in vivo proliferative capacity, a single infusion achieved deep and durable responses (mFU = 1.4 months; ORR = 100%, *n* = 15; CR = 30% at DL1 and 40% at DL2; MRD negativity 100% at a sensitivity of 10^−5^), together with a favorable safety profile characterized by predominantly low‐grade CRS and absence of NE or ICANS [244]. In a CD38/BCMA dual‐target trial (ChiCTR1800018143; *n* = 23), ORR was 87%, sCR 52%, MRD 100%, and mPFS 17.2 months [230]. A phase I CS1/BCMA study (NCT04662099; *n* = 16) achieved 100% ORR, 46% sCR, and universal MRD negativity. The one‐year PFS and OS were 56.3% and 72.7%. Only 38% of patients experienced CRS, and no cases of ICANS were observed [231]. In addition to BCMA, novel antigens such as GPRC5D are under evaluation. A meta‐analysis of GPRC5D CAR‐T studies showed 87% ORR, 48% sCR, and 65% MRD negativity. CRS occurred in 83% of cases, with grade ≥3 CRS in 5% [245]. At the 30th European Haematology Association meeting, CARsgen reported phase I data for autologous GPRC5D CAR‐T product CT071 (NCT06407947). This treatment achieved an ORR of 100%, with 7 patients (70%) reaching sCR. In terms of safety, all responders experienced grade 1 CRS only; no DLT, ICANS or treatment‐related deaths occurred.

### 4.2 Solid Tumour

Compared with haematological malignancies, solid tumours present greater therapeutic challenges. Currently, curative surgery remains the cornerstone for most early‐stage solid tumours. Nevertheless, microscopic residual disease frequently persists post‐operatively, and complete eradication is rarely achieved; these residual nests constitute the principal driver of tumour recurrence [246]. Radiotherapy, chemotherapy and hyperthermia provide modest benefit but are limited by rapid emergence of resistance and recurrence [247, 248, 249, 250, 251, 252, 253]. Similarly, conventional targeted agents are hampered by the pronounced molecular heterogeneity of solid tumours [254]. Consequently, CAR‐T cell–based immunotherapy is being actively investigated as a novel strategy against solid tumours (Table 5).

**TABLE 5:** CAR‐T cell therapies for solid tumours entering clinical trials.

Target	Indication	Treatment	Pts. number	Efficacy	Safety	Phase	Registration	Ref.
**B7‐H3/ CD276**	**Recurrent glioblastoma (rGBM)**	Autologous B7‐H3 CAR‐T (TX103)	13	OS (12‐month): 83.3% (DL = 2 × 10^7^ cells, 6 × 10^7^ cells) mOS: 20.3 months (not reached) PR and CR: 2/3 (DL = 6 × 10^7^ cells)	DLTs: None Treatment‐related deaths: None TRAEs: mostly Grade 1–2, 3 Grade 3 (↑ICP, epilepsy, ↓consciousness)	I	NCT05241392	[255]
**CD44‐CD133**	**rGBM**	Tandem CD44‐CD133 armoured with a truncated IL7Ra intracellular domain CAR‐T cells (Tris‐CAR‐T)	4	Radiographic and symptomatic response observed in multiple patients Some showed marked tumour regression Long‐term tumour control pending FU	NT/ICANS: None AEs: None Grade >1 Well tolerated across all doses	I	NCT05577091	[256]
**CD70**	**Advanced clear cell renal cell carcinoma (ccRCC)**	Allogeneic CD70 CAR‐T cell (ALLO‐316)	38	ORR (CD70^+^, *n* = 30): 20% ORR (CD70 ≥50%, phase 1b, *n* = 9): 33% mFU: 6.8 months (0.8‐39.5) All responses are ongoing	DLTs: 2 (autoimmune hepatitis, cardiogenic shock) CRS: 2% Grade ≥3 ICANS: 0 Grade ≥3 TEAEs: 84% Grade ≥3, 3 Grade 5 GvHD: None	Ia/Ib	NCT04696731	[257]
**CD133**	**HCC**	CD133 CAR‐T cells	21	PR: 4.8% SD: 66.7% mPFS: 6.8 months mOS: 12 months	DLTs: None Hyperbilirubinaemia: 4 Grade 3 (pre‐existing biliary obstruction) Transient haematologic toxicity: 3–5 days post‐infusion, resolved <1 wk	II	NCT02541370	[26]
**CEA**	**Unresectable locally advanced or metastatic solid tumours**	Logic‐gated CEA‐targeting Tmod CAR‐T cells (A2B530)	14	PR (PANC, *n* = 3): 1 Evidence of possible dose‐dependent CAR‐T expansion	DLTs: None CRS: 1 (DL = 2 × 10^8^ cells, experienced possible Grade 2) NT/ICANS: None AEs: None Grade >3 Gastrointestinal AEs: Mostly early‐onset, Grade 1–2	I/II	NCT05736731	[258]
**CLDN6**	**CLDN6+ R/R solid tumours**	CLDN6 CAR‐T cells administered alone or in combination with a CAR‐T cell amplifying mRNA vaccine (CARVac)	22	ORR (*n* = 21 evaluable): 33% (1 CR) ORR (*n* = 7, Germ cell tumours at DL = 1 × 10^8^ cells): 57% DCR: 67% SD: 7	CRS: 10, 1 Grade 3 NT/ICANS: 1 Grade 1 DLTs: 2 (DL = 1 × 10^8^ cells, all resolved) CARVac well tolerated	I/II	NCT04503278	[160]
**CLDN18.2**	**CLDN18.2^+^ advanced gastric/gastro‐oesophageal junction cancer**	Autologous CLDN18.2 CAR‐T cells (CT041)	88	mPFS: 3.25 months	CRS: 95% AEs: 99% Grade ≥3	II	NCT04581473	[259]
	**GC/GEJ**	Autologous CLDN18.2 CAR‐T (IMC002)	16	ORR (*n* = 15): 66.7% DCR (*n* = 15): 93.3% CR (*n* = 15): 6.7%	DLTs, Treatment‐related death, ICANS, Grade ≥3 liver toxicity: None CRS: 100%, 75% Grade 1, 25% Grade 2 Grade ≥3 TRAEs: Mainly hematologic & lymphodepletion‐related	I	NCT05946226	[260]
**Delta‐like‐ ligand 3 (DLL3)**	**R/R SCLC/LCNEC**	DLL3 CAR‐T cells with dnTGFβRII	9	PR: 1 (DL = 2 × 10^6^ cells/kg, *n* = 3) SD: 2(DL = 2 × 10^6^ cells/kg, *n* = 3), 3 (DL = 1 × 10^6^ cells/kg, *n* = 3) PD: 3 (DL = 0.3 × 10^6^ cells/kg, *n* = 3)	DLTs: None NT/ICANS: None CRS: 1 Grade 1 TEAEs: Grade≥3 (anaemia, leukopenia, neutropenia, all LD‐related)	I	NCT05680922	[261]
**EGFR**	**AR/RR NSCLC**	PiggyBac‐derived EGFR CAR‐T cells	9	PR: 1 (>13 months) SD: 6 PD: 2 mPFS: 7.13 months mOS: 15.63 months	CRS: None AEs: None Grade ≥4	I	NCT03182816	[262]
**EpCAM**	**EpCAM^+^ advanced gastric cancers**	Autologous EpCAM CAR‐T (IMC001)	12	ORR: 30% DCR: 70% mPFS: 4.5 months OS: 8.4 months PR: 33.3% (3 × 10^5^ cells/kg), 40% (1 × 10^6^ cells/kg)	TRD, SAE: None CRS: 5 Grade 3–4 Immune‐related hepatitis: 2 Grade 3–4 TRAEs: Mainly lymphopenia related to lymphodeleption	I	ChiCTR2100047129	[263]
	**Epithelial tumours**	EpCAM‐CAR‐T	12	PFS: 3 pts.>23 months SD: 5 PD: 8	Self‐limited AEs: 50% Grade 1/2 Leukopenia: 8.3% Grade 3 (reversible)	I	NCT02915445	[264]
**Guanylate cyclase‐C (GCC19)**	**Metastatic colorectal cancer (mCRC)**	GCC19 CAR‐T	15	PR: 40% (3 unconfirmed) PFS: 6.0 months (DL = 2 × 10^6^ cells/kg); 1.9 months (DL = 1 × 10^6^ cells/kg); P = 0.03 SD: 5 mOS: 22.8 months (95% CI, 13.4–26.1) CBR: 73% mFU: 22.8 months (range 3.7–35.7)	CRS: 93%, 87% Grade 1, 8% Grade 2 NT/ICANS:1 Grade 4 (resolved within 30 days with high‐dose corticosteroids) AEs: 93% Grade ≥3 Treatment‐related deaths: None Diarrhoea: 93%; 6 Grade 1–2, 8 Grade ≥3 Dasatinib: 3 Infliximab: 11 Haematologic AEs: 100% (transient, mostly Grade 1–2) Lymphocytopenia: Reversible within 3 months	I	ChiCTR2000040645	[25]
**GD2**	**R/R, high‐risk neuroblastoma**	3^rd^‐generation GD2 CAR‐T cells (GD2‐CART01)	27	ORR: 63% CR: 9 pts. PR: 8 pts. OS (3 years): 60% EFS: 36%	CRS: 74% Grade 1–2 (95% mild, 1 rapid clearance) Haematologic AEs: 100% Severe Transient hepatic toxicity: 7 Grade 3	I/II	NCT03373097	[265]
	**Paediatric and young adult GD2+ osteosarcoma and neuroblastoma**	GD2 CAR‐Ts (GD2‐CAR.OX40.28.z.iC9)	13	SD (Day 28): 83.3% (30% SD remained stable at Day 60; all later progressed) PD (Day 28): 16.7%	DLTs: No CRS: 15.4% Grade 1 NT/ICANS: None	I	NCT02107963	[266]
**GPC3**	**Paediatric and adult R/R liver cancers**	GPC3 CAR‐T	6	Produced no objective antitumour responses and reached peak expansion at 2 weeks	Safe	I	NCT02932956; NCT04377932; NCT02905188; NCT05103631	[267]
		GPC3 CAR‐T cells co‐expressed IL‐15	12	ORR 33% DCR: 66%	CRS: ↑ incidence (controlled)			
	**Advanced HCC**	Autologous GPC3 TGFβRIIDN armoured CAR‐T	24	ORR (*n* = 22 evaluable): 50.0% DCR: 90.9% Tumour reduction: 90.9%	CRS: 91.7%, 1 Grade 3 NT/ICANS: None AEs (Grade ≥3): Reversible, 100% lymphocytopenia, 70.8% Neutropenia, 37.5% Thrombocytopenia, 16.7% ↑ Transaminase	I	NCT05155189	[268]
**Guanylyl cyclase 2C (GUCY2C)**	**mCRC**	GUCY2C CAR‐T cells (IM96)	20	ORR: 26.3% DCR: 73.7% mPFS: 7 months mDOR (DL = 12 × 10^8^ cells): 10 months	DLT or MTD: No CRS: 80% Grade 1–2, 1 Grade 3 (with Neurotoxicity) Diarrhoea: 55% Grade 3 Rash: 70% Grade 1–3 Oral mucositis: 35% Grade 1–3	I	NCT05287165	[269]
**HER 2**	**Advanced sarcoma**	autologous HER2 CAR‐T cells	14	CR: 21% SD: 29% PD: 50% mOS: 8.2 months (*n* = 13, 95% CI: 4.4–17) mPFS: 2.4 months (*n* = 14, 95% CI: 1.3–8.7) mFU: 8.2 months (range: 1.3–70.6 months)	CRS: 8 NT/ICANS: None Cytopenia (*n* = 13): 100% Grade 4	I	NCT00902044	[270]
**IL‐13Rα2**	**Recurrent high‐grade glioma (rHGG), mainly rGBM**	IL‐13Rα2 CAR‐T cells	65	CR (*n* = 58 evaluable): 2 SD or better (*n* = 58 evaluable): 50% PR (*n* = 58 evaluable): 2 mOS: 8 months	DLTs: No CRS: None AEs: 35% Grade ≥3 (1 encephalopathy, 1 ataxia) Transient cerebral oedema: 2 Grade 4	I	NCT02208362	[271]
**MSLN**	**Advanced malignant pleural mesothelioma (MPM)**	Non‐viral aPD1‐MSLN JL‐Lightning CAR‐T cells (BZT2312)	4 (DL = 0.5‐0.6 × 10^6^ cells/kg)	PR: 25% DCR: 75%	DLT: No CRS: 25% Grade 1 NT/ICANS: None	I	NCT06249256	[272]
			3 (DL = 0.8‐1.0 × 10^6^ cells/kg)	ORR: 100% CR:1 (at 3 months, maintained >9 months)	CRS: 2/3 Grade 3–4 Immune‐mediated pneumonia: 2/3 Grade 3 (manageable) Haematologic AEs: 100% Grade 3–4 (fully reversible with supportive care)			
	MSLN^+^ HLA‐A*02^−^ recurrent unresectable LA/mM cancer (OVCA, PANC, NSCLC)	Autologous logic‐gated Tmod CAR‐T cells (A2B694)	5	Efficacy‐evaluable: 5 pts. Peripheral blood: Positive in all pts. post‐infusion	DLTs: No CRS: None NT/ICANS: None Lymphopenia: 14.6% Decreased appetite: 12.5%	I/II	NCT06051695	[273]
**MUC1**	**Recurrent/metastatic breast cancer (R/M BC)**	CRISPR‐Cas9–edited PD‐1^KO^ MUC1‐targeted CAR‐T cells	12	SD: 41.7% Preliminary evidence of disease control Response associated with higher CAR^+^ copies on day 7	DLTs: No CRS or NT/ICANS: None Grade 3–5 AEs: 58.3% ↑AST, 50% fever, 41.7% ↑ALT Lymphopenia (transient): 1 Grade 4 SAEs or treatment discontinuation: None	I	NCT05812326	[274]
**PSCA**	**Metastatic Castration‐resistant Prostate Cancer and Metastatic Prostate Cancer**	PSCA‐targeted CAR‐T cells (BPX‐601)	33	PR: 2 (1 unconfirmed, mCRPC) PSA50 (mCRPC): 56% CAR‐T expansion & tumour infiltration observed	DLTs: 4 (DL = 5 × 10^6^ cells/kg) CRS: 12.5% mPDAC (4.2% Grade≥3), 100% mCRPC (22.2% Grade≥3) ↑ Cytokine with rimiducid Study terminated before RP2D	I	NCT02744287	[275]
**PSMA**	**mCRPC**	Autologous CAR‐T cells with dnTGFβR	13	mPFS: 4.4 months PSA30 response: 30.1% mOS: 15.9 months	CRS: Grade 1–2 common (Cohort 3), 2 Grade 3 (w/o LD), 1 Grade 4 (sepsis‐related death, DLT) AEs (Cohort 3): None Grade≥3	I	NCT03089203	[276]
**ROR1**	**TNBC/NSCLC**	Autologous ROR1 CAR‐T cells	18	ORR: 5.5%	DLT: 1 (DL = 1 × 107 cells/kg, NSCLC)	I	NCT02706392	[227]

Abbreviations: ALT, Alanine Aminotransferase; AR/RR, Advanced or Relapsed / Refractory; AST, Aspartate Aminotransferase; B7‐H3, B7 homolog 3 (CD276); CBR, Clinical Benefit Rate; ccRCC, Clear Cell Renal Cell Carcinoma; CEA, Carcinoembryonic Antigen; CI, Confidence Interval; CLDN 6, Claudin 6; CLDN18.2, Claudin 18.2; GC/GEJ, Advanced gastric and gastroesophageal junction; DCR, Disease Control rate; DLL 3, Delta‐like‐ ligand 3; dnTGFβRII, Dominant‐negative TGF‐beta Receptor II; EpCAM, Epithelial Cell Adhesion Molecule; GD2, Disialoganglioside 2; GCC 19, Guanylate Cyclase‐C targeting CAR coupled with CD‐19 targeting; GPC3, Glypican‐3; GUCY2C, Guanylyl Cyclase C; IL‐15, Interleukin 15; LCNEC, Large Cell Neuroendocrine Carcinoma; LD, Lymphodepletion; MPM, Malignant Pleural Mesothelioma; MSLN, mesothelin; mCRC, Metastatic colorectal cancer; mCRPC, Metastatic Castration‐resistant Prostate Cancer; PANC, Pancreatic Cancer; PSA 50/30, Prostate‐specific Antigen Decline ≥50%/≥30%; PSCA, Prostate Stem Cell Antigen; PSMA, Prostate‐Specific Membrane Antigen; R/M BC, Recurrent/Metastatic Breast Cancer; rGBM, Recurrent Glioblastoma; rHGG, Recurrent High‐grade Glioma; ROR1, Receptor Tyrosine Kinase‐like Orphan Receptor 1; RP2D, Recommended Phase 2 Dose; SD, Stable Disease; SCLC, Small Cell Lung Cancer; TEAE, Treatment‐emergent Adverse Event; TTP, Time to Progression.

#### 4.2.1 Glioblastoma Multiforme

GBM is the most common and aggressive primary malignant brain tumour, representing 50% of malignant central nervous system tumours and occurring at ∼4.0 per 100,000 population annually [277]. Despite standard treatment strategies for GBM – including surgery, radiotherapy, and temozolomide‐based chemotherapy – the mOS was only 20.9 months, and virtually all patients relapse [278]. The high heterogeneity and immunosuppressive microenvironment of GBM pose major challenges to immunotherapy. However, in recent years, the CAR‐T studies in rGBM have yielded encouraging early signals. Early data show acceptable safety and potential survival benefit, particularly with novel targets such as B7‐H3 and IL‐13Rα2. In a Phase I clinical trial (NCT05241392) targeting B7‐H3 (CD276), 13 patients with rGBM received treatment, among whom two patients in the DL2 group (60 million cells) achieved PR or CR. In addition, twelve‐month OS was 83% in both dose levels, and mOS was not reached. Moreover, the treatment was well tolerated, with no DLTs or treatment‐related deaths; three grade 3 events (intracranial pressure, seizures, reduced consciousness) occurred [255]. In another Phase I clinical trial (NCT02208362) targeting IL‐13Rα2, 65 patients with advanced gliomas, primarily rGBM, were enrolled. Among the 58 evaluable patients, 2 achieved CR and 2 achieved PR, with an overall disease control rate of 50%. In addition, no CRS or DLTs were observed, although 35% of patients experienced Grade ≥3 adverse events, including transient cerebral oedema and central nervous system symptoms [271].

#### 4.2.2 Carcinoma of the Colon and Rectum

CRC is the third most common cancer and the second leading cause of cancer‐related deaths worldwide [279]. Metastatic CRC carries a 5‐year overall survival rate of ∼15%, and targeted or immune therapies offer limited benefit [280]. In a GCC19 CAR‐T study (ChiCTR2000040645), 15 patients with mCRC were enrolled. In the high‐dose group, the PFS reached 6 months, significantly longer than in the low‐dose group. In addition, the overall CBR was 73%, with a PR rate of 40% and a mOS of 22.8 months. CRS occurred in 93% (mostly grade 1); one grade 4 neurotoxicity resolved with therapy, while diarrhoea occurred in 93% of patients. Moreover, reversible lymphopenia and mild‐to‐moderate haematological toxicity were observed, with no treatment‐related deaths [25]. In another Phase I trial of GUCY2C CAR‐T cells (NCT05287165, *n* = 20), the ORR was 26.3%, the DCR was 73.7%, and the mPFS was 7 months. In the highest dose group, the mDOR was 10 months. CRS occurred in 80% of patients, mostly Grade 1–2, with one case of Grade 3 CRS accompanied by neurotoxicity. Main adverse events were grade 3 diarrhoea (55%), rash (70%), and oral mucositis (35%) [269].

#### 4.2.3 Breast Cancer

Since 2020, BC has been the most common malignancy and the leading cause of cancer death in women worldwide [281]. Early‐stage BC is often curable with surgery and adjuvant therapy, yet R/M disease remains difficult to treat [282]. With the deepening understanding of TAAs, mucin‐1 (MUC1) has emerged as a promising CAR‐T target due to its overexpression and tumour specificity across multiple BC subtypes. In a Phase I clinical trial (NCT05812326, *n* = 12, R/M BC), SD occurred in 42%, correlating with higher CAR‐T levels on day 7, suggesting disease‐control potential. In addition, the treatment was well tolerated with no DLTs and no grade ≥3 CRS or neurotoxicity. Moreover, the most frequent adverse events were elevated AST (58%), fever (50%), and elevated ALT (42%); one grade 4 transient lymphopenia occurred [274].

#### 4.2.4 NSCLC

Non‐Small Cell Lung Cancer (NSCLC) constitutes ∼85% of lung cancers and is the leading cause of cancer death in men worldwide [283]. Despite great advances in targeted therapy and immunotherapy, treatment options remain limited and prognosis poor for patients with AR/RR NSCLC [284]. Candidate CAR‐T targets include MUC1, CEA, HER2, MSLN, ROR1, GPC3, EGFR, and PD‐1 [285]. In a phase I EGFR CAR‐T study (NCT03182816; *n* = 9, AR/RR NSCLC), the PR rate was 11%, with responses lasting >13 months. mPFS and mOS were 7.1 and 15.6 months, respectively. In addition, no CRS or grade ≥4 events were observed, indicating that the therapy was well tolerated [262].

#### 4.2.5 Hepatocellular Carcinoma

HCC is the sixth most common cancer and the third leading cause of cancer death worldwide [286]. While early‐stage HCC may be treated with surgical resection or local therapies, most cases are diagnosed at advanced stages, and effective systemic options remain scarce. Although targeted therapies and immune checkpoint inhibitors confer a modest survival benefit, the ORR remains low [287]. Investigational CAR‐T targets include GPC3, EpCAM, claudin‐18.2, CD147, EGFRvIII, c‐MET and death receptor‐5 (DR5) [247]. In a phase II CD133 CAR‐T study (NCT02541370; *n* = 21), PR was 5%, whereas 67% achieved SD. In addition, the mPFS and mOS were 6.8 months and 12 months, respectively. In terms of toxic side effects, hyperbilirubinaemia occurred chiefly in patients with biliary obstruction; transient haematological toxicity resolved within one week, and no DLTs were reported [26].

#### 4.2.6 Prostate Cancer

Prostate cancer (PC) is the second most common malignancy in men worldwide. Among its subtypes, Metastatic castration‐resistant PC carries a mOS of ∼3 years despite endocrine therapy and chemotherapy [288, 289]. For treating PC patients, CAR‐T cell therapy has mainly focused on TAAs such as PSCA PSMA, prostate‐specific antigen (PSA), and EpCAM [290]. A phase I trial of BPX‐601 GoCAR‐T cells targeting PSCA (NCT02744287; *n* = 33, metastatic PC/mCRPC), CAR‐T cells expanded peripherally and infiltrated tumours; two mCRPC patients achieved PR, and 56% attained PSA50. Unfortunately, four DLTs occurred at the highest dose, and the study closed early after the second DLT without establishing an RP2D [275]. In another phase I trial (NCT03089203; *n* = 13, mCRPC), PSA30 was achieved in 30%, and the mPFS and mOS were 4.4 and 15.9 months, respectively. In addition, although CRS occurred, it was predominantly grade 1–2 [276].

### 4.3 Autoimmune Disease

While CAR‐T therapy has demonstrated its transformative potential in haematological malignancies and is now being actively explored in solid tumours, its dynamic immune reprogramming capabilities are prompting a fundamental expansion of its therapeutic scope beyond oncology [291, 292]. Increasingly, researchers are leveraging the precise, powerful, and persistent nature of CAR‐T cells to address a new class of diseases: autoimmune disorders [292]. AIDs arise from immune system dysregulation, leading to abnormal activation of autoreactive T and B cells and the production of autoantibodies that attack normal tissues, resulting in chronic inflammation and organ damage [293]. Common therapeutic approaches include immunosuppressants (such as glucocorticoids and conventional immunosuppressive agents), biologics, and targeted small‐molecule drugs like JAK inhibitors. Although these therapies provide certain efficacy, they carry risks of serious side effects. For example, TNF‐α inhibitors increase the risk of infections, including a 3‐ to 4‐fold elevated risk of tuberculosis reactivation, and are associated with a higher incidence of malignancies, such as a 2.02‐fold increased risk of melanoma; long‐term use may also elevate autoantibody levels and increase the risk of deep vein thrombosis [294, 295, 296, 297]. Therefore, there is an urgent need for improved therapeutic strategies.

#### 4.3.1 Classification of Strategies for CAR‐T Therapy in Autoimmune Diseases

Preclinical exploration of CAR‐T therapy for AIDs began in 2002, when Geiger and colleagues engineered T cells specific for myelin basic protein and suppressed disease in experimental autoimmune encephalomyelitis, a murine model of multiple sclerosis. Their study eliminated autoreactive T cells and skewed immunity toward a Th2 phenotype, establishing the concept of CAR‐mediated antigen‐specific tolerance [298]. Subsequent approval of Tisagenlecleucel (Kymriah) confirmed that CD19‐directed CAR‐T cells efficiently deplete B cells and reset autoreactive circuits, providing a new option for B cell–mediated AIDs.

The application of CAR‐T therapy in AIDs primarily follows two major strategies (Figure 6): First, cytotoxic CAR‐T cells are retargeted – via gene editing – to B cell antigens such as CD19 or BCMA, rapidly ablating autoantibody‐producing cells. This approach is applicable to antibody‐mediated diseases such as SLE and myasthenia gravis [299]. Second, immunomodulatory approaches employ CAR‐engineered regulatory T cells (CAR‐Tregs) that home to inflamed tissues and suppress immunity, or chimeric autoantibody receptor T cells (CAAR‐T) that display self‐antigens and delete cognate autoreactive B cells, delivering antigen‐specific tolerance without systemic suppression [300, 301].

**FIGURE 6:**
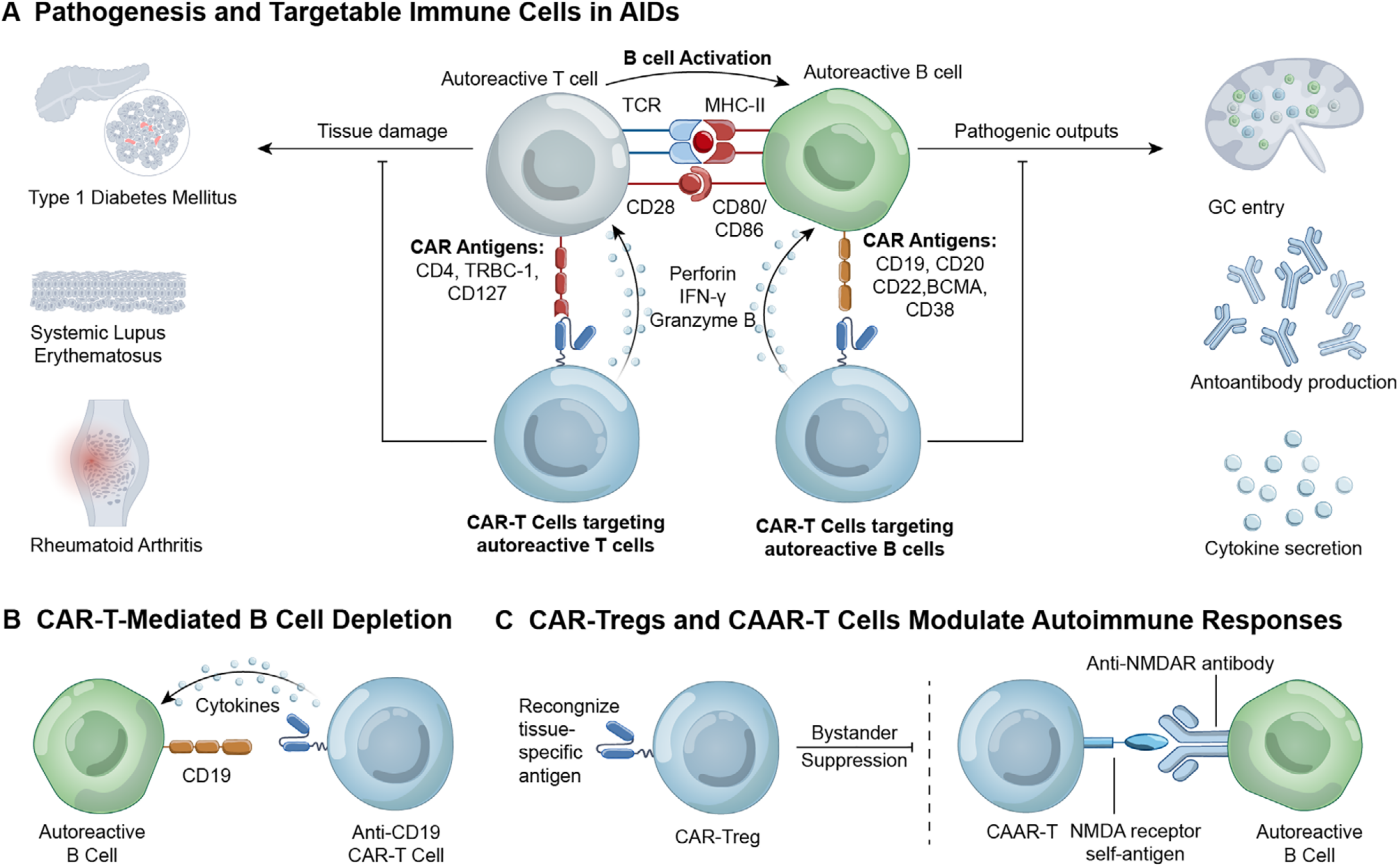
Strategies for CAR‐T therapy in AIDs. (A) Pathogenic autoreactive T cells and B cells drive AIDs, including Type 1 diabetes mellitus, SLE, and rheumatoid arthritis. Activated B cells produce autoantibodies and pro‐inflammatory cytokines. CAR‐T cells are engineered to target either autoreactive T cells or autoreactive B cells. (B) Strategy 1: Anti‐CD19 CAR‐T cells directly eliminate autoreactive B cells, reducing autoantibody and cytokine production. (C) Strategy 2: CAR‐Treg cells suppress immune responses by recognizing tissue‐specific antigens and bystander suppression; CAAR‐T cells target and deplete autoreactive B cells expressing specific autoantibodies.

#### 4.3.2 Application of CAR‐T Therapy in the Treatment of Autoimmune Disease

Studies show that complete B cell depletion correlates with superior outcomes; patients achieving full depletion respond better than those with partial depletion [302]. Given the potent B cell–depleting capacity of CAR‐T cells in haematologic cancer, this platform is now viewed as a promising strategy for AIDs. In SLE models, CD19 CAR‐T cells eradicated B cells in blood and secondary lymphoid organs, maintaining depletion for > 6 months and markedly suppressing disease [303, 304]. In addition, lupus nephritis (LN) was markedly alleviated, with reduced proteinuria levels, improved renal histopathology, and slow B cell repopulation, suggesting durable remission. Importantly, no serious side effects were observed, supporting a favourable in vivo safety profile [303, 304]. These preclinical findings underscore the efficacy and safety of CD19 CAR‐T therapy and further support its advancement into clinical applications.

The first clinical use of CD19 CAR‐T cells in autoimmunity was reported in 2021 in a 20‐year‐old patient with refractory SLE, active LN, nephrotic syndrome and arthritis [305]. Prior to CAR‐T treatment, the patient had received multiple immunosuppressive agents (including hydroxychloroquine, corticosteroids, and tacrolimus), as well as B cell‐targeted therapies (belimumab and rituximab), yet the disease remained uncontrolled. Following CD19 CAR‐T cell infusion, a rapid expansion of CAR‐T cells was observed, rising from 0.3% of circulating T cells on day 3 to 28% on day 9. Subsequent monitoring over a seven‐week period showed a gradual and physiological decline in CAR‐T cell levels. Within one week, circulating B cells were depleted; by week 5, anti‐dsDNA titres had fallen by ∼99%. Concomitantly, SLE manifestations resolved. The SLE Disease Activity Index (SLEDAI) dropped from 16 to 0, indicating CR with no signs of active inflammation or organ damage. Notably, no CRS or ICANS occurred. These findings provide further clinical evidence supporting the safety and efficacy of CD19 CAR‐T therapy and highlight its promising potential in treating AIDs by targeting pathogenic B cells [305].

In addition, recent work has extended CD19 CAR‐T therapy to severe AIDs [306]. A first‐in‐human study (NCT05859997) used CRISPR‐edited allogeneic CD19 CAR‐T cells to treat refractory immune‐mediated necrotizing myopathy and diffuse cutaneous systemic sclerosis. In three recipients, off‐the‐shelf CAR‐T cells persisted >3 months and fully depleted B cells within two weeks. During the six‐month FU, no CRS or other severe adverse events were observed. Clinical assessments revealed improvements in disease scores, while histopathological analyses indicated reversal of inflammation and fibrosis, suggesting that CAR‐T therapy may induce a durable remission in these refractory autoimmune conditions [306].

However, CD19 CAR‐T therapy primarily targets B cells and may fail to eliminate long‐lived plasma cells responsible for sustained autoantibody production, potentially leading to persistent antibody levels and an increased risk of relapse [307]. To address this limitation, researchers have developed BCMA‐CD19 dual‐target CAR‐T cells (cCAR‐T), which simultaneously target both B cells and long‐lived plasma cells, thereby achieving a deeper immune reset and broadening the therapeutic potential of CAR‐T therapy in SLE and LN. A Phase I clinical trial was conducted to evaluate the safety and immune resetting capacity of cCAR‐T in patients with SLE/LN. Results demonstrated that within three months, autoantibodies – including those produced by long‐lived plasma cells – were completely cleared, complement levels returned to normal, and patients maintained symptom remission and drug‐free status for up to 46 months of follow‐up. In addition, SLEDAI‐2000 scores decreased from 10.6 to 2.7 at 3 months, and renal function significantly improved within 90 days in all 10 LN patients. Only mild CRS was observed, with no severe adverse events, indicating a favourable safety profile and good tolerability [307].

In the strategy of re‐establishing immune tolerance through engineered T cells, preclinical studies have primarily focused on CAR‐Tregs and CAAR‐T. CAR‐Tregs target inflammation‐associated antigens, allowing them to migrate selectively to diseased tissues. There, they can locally suppress inflammation by secreting inhibitory cytokines such as IL‐10 and TGF‐β, or by directly inhibiting effector T cell activation.Evidence supporting the therapeutic potential of antigen‐specific regulatory T cells has also emerged from studeis of collagen type II (ColII)‐specific Tr1 cells (Col‐Tregs). In the collagen antibody‐induced arthritis (CAIA) model, mice received a single infusion of collagen type II (ColII)‐specific Tr1 cells (Col‐Tregs; 1 × 10^6^ cells/mouse) targeting type II collagen (Col II) [308]. Results showed that Col‐Tregs accumulated in joints and draining lymph nodes (DLNs), significantly reduced arthritis scores, and, although not completely preventing disease progression, effectively delayed its onset. In the collagen‐induced arthritis (CIA) model of chronic inflammation, Col‐Tregs demonstrated dose‐dependent efficacy: the high‐dose group (3 × 10^6^ cells/mouse) exhibited significantly lower arthritis scores and incidence rates compared to the low‐dose (1 × 10^6^ cells/mouse) and control groups. Long‐term safety assessments revealed that 2–4 months after infusion, only 1 out of 17 mice exhibited weak Col‐Treg signals in the axillary lymph nodes, with no signs of abnormal proliferation or tumourigenicity. These findings confirm local‐targeting advantages and an acceptable safety profile, highlighting the need for dose optimization and early intervention [308]. Although Col‐Tregs are not CAR‐engineered, they support antigen‐directed regulatory T‐cell therapy for autoimmune arthritis.

CAAR‐T cells function by displaying self‐antigens – such as Desmoglein 3 (Dsg 3) in the case of pemphigus vulgaris (PV) – on their surface to specifically recognize and eliminate B cells carrying the corresponding autoantibodies, while sparing normal B cells and thus avoiding broad immunosuppression [309]. PV is an autoimmune disease mediated by anti‐Dsg3 autoantibodies. Researchers designed a CAAR construct using Dsg3 as the extracellular domain and 4‐1BB‐CD3ζ as the intracellular signalling domain, enabling T cells to selectively eliminate Dsg3‐specific B cells and thereby remove the source of pathogenic autoantibodies. Preclinical studies demonstrated that DSG3 CAAR‐T cells could effectively deplete anti‐Dsg3 B Cell Receptor (BCR^+^) B cells in PV‐related models, showing comparable efficacy to anti‐CD19 CAR‐T cells. In vitro, DSG3 CAAR‐T cells efficiently lysed Nalm‐6 B cells expressing anti‐Dsg3 BCR but exhibited no cytotoxicity toward B cells lacking Dsg3 BCR expression, confirming their specificity. In a PV mouse model (NSG mice engrafted with PV hybridomas AK18/AK19/AK23), DSG3 CAAR‐T cells suppressed hybridoma growth, reduced anti‐Dsg3 autoantibody levels, and prevented oral mucosal blistering. Notably, even when treatment was initiated during the exponential growth phase of target cells, DSG3 CAAR‐T cells retained the capacity to effectively eliminate anti‐Dsg3 BCR^+^ B cells. Safety evaluations revealed that DSG3 CAAR‐T cells could survive long‐term in the absence of antigen stimulation without being depleted by soluble autoantibodies, and no off‐target toxicity was detected – supporting clinical translation [309].

In recent years, both CAAR‐T and CAR‐Tregs have shown promising potential in preclinical studies. Animal models and in vitro experiments have demonstrated that these therapies can effectively eliminate self‐antigen–specific B cells or modulate immune tolerance, offering novel therapeutic approaches for AIDs. However, these strategies remain in the early stages of clinical investigation, and the results of related clinical trials have yet to be published. Currently, several clinical trials are underway. For example, the DSG3‐CAAR‐T trial is evaluating its application in PV (NCT04422912), aiming to suppress PD by specifically targeting Dsg3 autoantibody‐producing cells. Additionally, QEL‐001, an autologous CAR‐Tregs developed for HLA‐A2 mismatched liver transplant recipients, is being assessed for its safety and tolerability (NCT05234190), with the goal of preventing transplant rejection following the withdrawal of immunosuppressive drugs. Taken together, these studies are designed based on encouraging preclinical data, and investigators hope to further validate the clinical efficacy and long‐term safety of CAAR‐T and CAR‐Tregs.

## 5 Conclusion

CAR‐T cell therapy has revolutionized the treatment landscape of haematological malignancies and is rapidly expanding into the fields of solid tumours and AIDs. In blood cancers, it achieves durable remission through the precise eradication of malignant cells, while in AIDs, it opens novel therapeutic avenues by selectively depleting autoreactive immune cell populations. However, several major obstacles remain, including tumour heterogeneity, the immunosuppressive TME, functional exhaustion of T cells, antigen escape, high manufacturing costs and limited accessibility, as well as unique toxicities such as neurotoxicity and haematologic toxicity. These challenges underscore the necessity of balancing efficacy and safety across different disease contexts. Future advances will rely on precision engineering and adaptive regulation. Gene‐editing platforms such as CRISPR/Cas9 are enabling the development of more potent and safer CAR‐T products, while logic‐gated and switchable CAR designs promise greater controllability and specificity. Strategies to remodel the TME can further enhance CAR‐T persistence under hostile conditions. Meanwhile, allogeneic CAR‐T products and in vivo delivery technologies are reducing production costs and expanding accessibility. Artificial intelligence and deep learning are progressively integrated into key aspects of CAR‐T research, including multi‐omics dimensionality reduction, modelling CAR‐T–tumour interactions, molecular docking and protein structure prediction, side‐effect management, and the optimization of personalized therapy [310]. Collectively, these innovations are shaping CAR‐T therapy into an intelligent, programmable immunotherapy platform, with the potential to evolve from a specialized intervention into a broadly applicable and transformative clinical paradigm.

## Author Contributions

R.Y., Y.Y., and W.G.W. jointly conceptualized and supervised the review, designed the overall structure, integrated perspectives across different sections, critically revised the manuscript, and approved the final version for submission. Y.S.B. coordinated the project and was responsible for conceptualization, writing (original draft, review and editing), and initial figure visualization. J.T.Q. contributed to conceptualization, writing (original draft, review and editing), and initial figure visualization. Z.J.D. contributed to conceptualization, writing (original draft, review and editing), and figure visualization (both initial and final versions). All authors have meticulously read the final manuscript and given their approval for its publication.

## Conflicts of Interest

The authors declare no conflicts of interest.
